# Therapeutic Targeting of miR-21 Restores SASH1 and Sensitizes HBV-HCC to Sorafenib

**DOI:** 10.3390/cancers18061038

**Published:** 2026-03-23

**Authors:** Kyuyoung Han, Eun-Kyoung Jwa, Suhyeon Ha, Jiye Kim, Ryunjin Lee, Eunkyeong Lee, Seoon Kang, Hye Ok Kim, Hyunhee Kwon, Dong-Hwan Jung, Young-In Yoon, Gi-Won Song, Gil-Chun Park, Tae Won Kim, Jung-Man Namgoon, Shin Hwang, Eunyoung Tak, Sung-Gyu Lee

**Affiliations:** 1 Department of Oncology, Asan Medical Center, University of Ulsan College of Medicine, Seoul 05505, Republic of Korea; 2 Division of Hepatobiliary Surgery and Liver Transplantation, Department of Surgery, Asan Medical Center, University of Ulsan College of Medicine, Seoul 05505, Republic of Korea; 3 Department of Pediatric Surgery, Asan Medical Center Children’s Hospital, University of Ulsan College of Medicine, Seoul 05505, Republic of Korea; 4 Asan Institute of Life Sciences, Asan Medical Center, University of Ulsan College of Medicine, Seoul 05505, Republic of Korea; 5 Department of Medicine, College of Medicine, Kyung Hee University, Seoul 02447, Republic of Korea; 6 Division of Liver Transplantation and Hepatobiliary Surgery, Department of Surgery, Asan Medical Center, University of Ulsan College of Medicine, Seoul 05505, Republic of Korea; 7 Asan Preclinical Evaluation Center for Cancer TherapeutiX, Asan Medical Center, Seoul 05505, Republic of Korea; 8 Department of Surgery, Asan Medical Center, University of Ulsan College of Medicine, Seoul 05505, Republic of Korea

**Keywords:** miR-21, SASH1, HBV-associated hepatocellular carcinoma, Sorafenib resistance, PI3K/AKT/mTOR signaling

## Abstract

Background: Sorafenib resistance remains a major barrier to effective therapy in hepatitis B virus (HBV)-associated hepatocellular carcinoma (HCC). Introduction: Here, we identified a previously undefined mechanism by which miR-21 promotes sorafenib resistance by suppressing the tumor suppressor SASH1 and enhancing HBx-driven PI3K/AKT/mTOR signaling. Methods: miR-21 expression was markedly elevated in HBV-HCC tissues, HBV-integrated HCC cell lines, and hypoxic conditions. Bioinformatic analyses and luciferase reporter assays confirmed SASH1 as a direct miR-21 target. Results: Mechanistically, SASH1 was functionally associated with HBx-related oncogenic signaling and influenced apoptotic responses. miR-21 inhibition reduced HBV-HCC cell proliferation, increased apoptosis, and restored sorafenib sensitivity in vitro. In an orthotopic HBV-HCC mouse model, the combined administration of miR-21 inhibitor and sorafenib elicited markedly greater tumor suppression and restoration of the SASH1 expression than either monotherapy did. Discussion: Therefore, these findings suggested that the miR-21/SASH1 pathway contributed to therapeutic resistance in HBV-associated HCC and highlighted that miR-21 targeting could be an efficient strategy to improve sorafenib response. Conclusions: The miR-21/SASH1 axis play a critical role in sorafenib resistance in HBV-associated HCC, and targeting miR-21 may provide a promising therapeutic strategy to enhance treatment efficacy.

## Simple Summary

Hepatitis B virus (HBV)-related hepatocellular carcinoma (HCC) frequently develops resistance to sorafenib, limiting treatment efficacy. We showed that miR-21 is highly expressed in HBV-positive HCC and directly suppresses the tumor suppressor SASH1. Loss of SASH1 enhances HBx-driven oncogenic signaling and reduces sorafenib-induced apoptosis. miR-21 inhibition restores the SASH1 expression and significantly improves sorafenib response in in vitro and in vivo models. These findings suggested that targeting miR-21 may represent a promising strategy to overcome sorafenib resistance in HBV-associated HCC.

## 1. Introduction

Hepatocellular carcinoma (HCC) remains a global health challenge, with a 5-year survival rate of only 31% [1]. Although the global burden of hepatitis B virus (HBV)-related hepatocellular carcinoma (HCC) has declined with the implementation of widespread vaccination programs, it remains a major cause of liver cancer in several regions, particularly in East Asia, including China and South Korea [2,3]. Notably, HCC risk is still high in cases where HBV DNA, particularly the region encoding the HBx protein, is integrated into the host genome [4,5]. Such integration can lead to HCC development despite antiviral therapy; these tumors often exhibit resistance to treatments such as sorafenib, thereby complicating therapeutic approaches [6]. Despite advances in treatment modalities, including chemotherapy, surgical resection, and liver transplantation, most patients present with an advanced disease and limited therapeutic options [7].

MicroRNAs (miRNAs) have emerged as crucial regulators of cellular processes, including those involved in cancer development and progression [8,9,10]. Among them, miR-21 has gained particular attention as a prominent oncomiR that promotes tumor proliferation by suppressing tumor suppressor genes [11,12,13,14,15]. Although miRNAs have been implicated in modulating sorafenib resistance in HCC, their specific roles in HBV-related HCC remain poorly understood.

Recent advances in cancer genomics have refined our understanding of HCC; as such, distinct molecular subgroups have been identified. However, the heterogeneity within these subgroups presents challenges to the development of targeted therapies [16]. Sorafenib, a multi-kinase inhibitor, is the standard first-line treatment for advanced HCC; it inhibits tumor cell proliferation and angiogenesis by targeting the Raf/MEK/ERK pathway and growth factor receptors [17]. However, its efficacy is limited to approximately 30% of patients with HCC, with frequent development of resistance and substantial side effects. The frequent development of sorafenib resistance and the molecular mechanisms underlying therapeutic responsiveness should be elucidated, and the involvement of miR-21 in tumor survival and resistance-associated signaling pathways should be described [18,19,20]. Therefore, we sought to investigate its potential contribution to sorafenib resistance in HBV-associated HCC.

This study aimed to elucidate the role of miR-21 in HBV-related HCC and its potential contribution to sorafenib resistance. We investigated the interaction between miR-21 and *SASH1*, a tumor suppressor gene, in the context of HBV-HCC. Our research explored how this mechanism might enhance sorafenib resistance and examined its relationship with the HBx protein, a characteristic component of HBV. We also provided new insights into the molecular mechanisms underlying HBV-HCC progression and therapy resistance by examining the miR-21 expression in HCC tissues and cell lines, exploring its influence on HCC cell survival, and demonstrating its effects on an orthotopic HCC xenograft model.

Furthermore, we presented evidence of a significant association between miR-21, SASH1 suppression, and the HBx protein in HBV-HCC. This study not only enhanced our understanding of HBV-related hepatocarcinogenesis but also suggested new therapeutic strategies, particularly in combination with existing treatments such as sorafenib, for this challenging subtype of HCC.

## 2. Materials and Methods

### 2.1. Human Liver Tissue Samples

Liver tissue samples were obtained from the Bio Resource Center (BRC) of Asan Medical Center (Seoul, Republic of Korea). HCC tissues and adjacent non-tumor tissues from eight patients (mean age, 56.5 years; range, 51–69 years) who underwent liver resection between November 2018 and January 2019 were used. The samples were quickly frozen in liquid nitrogen and stored until use. Patient information is presented in Table 1.

### 2.2. The Cancer Genome Atlas (TCGA) Data Analysis

The standardized miRNA-seq data of human liver HCC were downloaded from Broad TCGA GDAC Firehose (http://gdac.broadinstitute.org/). miR-21 expression levels in 50 adjacent non-tumor and 347 HCC samples were normalized and compared.

### 2.3. Cell Lines and Culture

HepG2 (Korean Cell Line Bank, Seoul, Republic of Korea; no. 88065) and HepG2.2.15 human HCC (kindly gifted by the Korean Advanced Institute of Science and Technology, Daejeon, Republic of Korea) cell lines were used. The cells were cultured in Dulbecco’s modified Eagle’s medium (DMEM; Hyclone, Logan, UT, USA) supplemented with 10% fetal bovine serum (FBS; Invitrogen Life Technologies, Waltham, MA, USA) at 37 °C in a humidified incubator containing 5% CO_2_.

### 2.4. Hypoxia Treatment

HCC cells were subjected to hypoxia (1% O_2_, 5% CO_2_, 94% N_2_) in a glucose and sodium pyruvate-free DMEM (Thermo Fisher Scientific, Waltham, MA, USA) in a multi-gas incubator (VS-2050CO; Vision Scientific, Daejeon, Republic of Korea) for 6 h.

### 2.5. Cell Transfection

The miR-21 inhibitor (Qiagen, Hilden, Germany) is a chemically synthesized single-strand RNA that specifically inhibits endogenous miR-21 function after transfection into cells. A scrambled oligo was obtained from Bioneer (Daejeon, Republic of Korea) and used as a negative control (NC). The cells were seeded in six-well plates and cultured to approximately 30% confluency before transfection. For each well, Lipofectamine^®^ 2000 was diluted in Opti-MEM (Thermo Fisher Scientific) in accordance with the manufacturer’s instructions. Briefly, diluted Lipofectamine^®^ 2000 was mixed with diluted 150 nM miR-21 inhibitor, 100 nM miR-21 mimic, or NC and then incubated at room temperature (RT) for 30 min with gentle agitation. The mixture was added to HCC cell culture plates and incubated at 37 °C in an atmosphere containing 5% CO_2_. After 48 h of incubation, the medium was discarded, and the cells were prepared for subsequent experiments. The sequences of siRNA and plasmid constructs used in this study are provided in Supplementary Table S3.

### 2.6. Dual-Luciferase Reporter Assay

The target genes and binding site of miR-21 were predicted by miRDB (http://mirdb.org/), miRWalk 2.0 http://zmf.umm.uni-heidelberg.de/apps/zmf/mirwalk2/miRretsys-self.html (accessed on 14 September 2020, and Target scan (http://www.targetscan.org/). SASH1 3′-UTR containing the wild-type (WT) or mutated (Mut) predictive miR-21 binding site was subcloned into a pmirGlo dual-luciferase miRNA target expression vector (Promega Corporation, Seattle, WA, USA) located at 5′ of the firefly luciferase. The HCC cells were seeded on a 48-well plate with 4.0 × 10^3^ cells per well and co-transfected with 1 μg of pRL-SV40, 1.5 μg of pmirGlo-SASH1 3′-UTR construct, 150 nM miR-21 inhibitor, 60 nM mimic, or NC. Transfection was performed using Lipofectamine^®^ 2000 at 37 °C for 6 or 48 h under normoxic or hypoxic conditions. The activities of firefly and *Renilla* luciferase were measured using a dual-luciferase reporter assay (Promega Corporation, Seattle, WA, USA). The activity of *Renilla* luciferase for each sample was normalized to that of firefly luciferase.

### 2.7. Cell Viability and Colony Formation Assays

The MTT assay was performed to detect cell viability. The cells were seeded in 48-well plates at a density of 4 × 10^3^ cells in 300 µL/wells and cultured for 24 h at 37 °C to allow them to adhere to the plate. After transfection, they were washed with phosphate-buffered saline (PBS), and 200 µL of MTT solution (1 mg/mL; Glentham, Corsham, UK) was added to each well. After incubation for 30 min, the solution was discarded, and 150 µL of DMSO (Glentham, Corsham, UK) was added to each well. Subsequently, the plates were agitated for 15 min to let the crystalized precipitates fully dissolve. Absorbance was measured at 540 nm by using a microplate reader (Sunrise; TECAN, Männedorf, Switzerland).

For colony formation assay, crystal violet (Sigma-Aldrich, Waltham, MA, USA) was dissolved in absolute ethanol and diluted with distilled water to prepare 0.2% crystal violet solution. HCC cells were seeded into 12-well plates at a density of 3 × 10^4^ cells and cultured at 37 °C for 24 h. After 72 h of transfection, the cells were removed from the medium and washed with PBS. They were fixed with 4% formaldehyde for 30 min and stained with 0.2% crystal violet solution for 15 min. The plates were washed with tap water. Images were taken by a camera, and the number of colonies was counted using the NIH ImageJ software.

### 2.8. Xenograft Orthotopic HCC Mouse Model

Eight-week-old male NOD-Rag_2_^−/−^Il2rg^−/−^ (NRG) mice (JOONGAH Bio, Suwon-si, Republic of Korea) were used. In this procedure, 1 × 10^6^ HepG2.2.15 HBV-HCC cells suspended in PBS with 10% Matrigel (Thermo Fisher Scientific) were injected into the left hepatic lobe. After 6 weeks, the mice were randomly divided into four groups and treated with saline (Control), sorafenib tosylate (sorafenib; 40 mg/kg daily by oral gavage; Bayer, Leverkusen, Germany), miR-21 inhibitor (inhibitor; 0.5 mg/kg, administered orally three times per week), or their combination (sorafenib + inhibitor) for 21 days. Body weights were measured daily, and the mice were sacrificed at the study endpoint. Humane endpoints were established as follows: mice losing 20% body weight or exhibiting pain-related behaviors. After the total liver weight that contained the xenografted tumor was measured, the liver tissues containing the HCC tumor mass were cut into several segments and immediately frozen with liquid nitrogen.

### 2.9. RNA Extraction and qRT-PCR

Total RNAs were extracted using QIAzol^®^ lysis reagent (Qiagen, Hilden, Germany) and RNeasy plus mini kit (Qiagen, Hilden, Germany) in accordance with a modified protocol. In brief, the cells or tissues were treated with 0.5 mL of Qiazol lysis buffer and mixed with 0.1 mL of chloroform. After centrifugation at the highest speed for 10 min, the clear supernatants were collected, and 70% ethanol was added. While the total mRNAs were bound on the silica column of RNeasy mini kit components, dissolved miRNAs in a collection tube were added with 100% ethanol and re-centrifuged in a new column. The samples were washed twice with RPE buffer (provided in the kit) and eluted with RNase-free distilled water.

The concentration and quality of the extracted RNAs were measured using Nanodrop2000 (Thermo Fisher Scientific). Reverse transcription was conducted using ReverTra Ace^®^ qPCR RT Master Mix (TOYOBO, Osaka, Japan) for an mRNA or miScript RT kit (Qiagen, Hilden, Germany) for miRNA. The transcripts were quantified via qRT–PCR by using the CFX Connect Real-Time PCR Detection System (Bio-Rad, Hercules, CA, USA). The 5× HOT FIREPol EvaGreen qPCR Supermix (Solis BioDyne, Tartu, Estonia) was used for mRNA, and the miScript SYBR Green PCR kit (Qiagen, Hilden, Germany) was used for miRNA. qRT–PCR was performed to analyze mRNA expression under the following conditions: initial activation step at 95 °C for 12 min, denaturation at 94 °C for 15 s, annealing at 55 °C for 30 s, and extension at 70 °C for 30 s. Otherwise, qRT–PCR for miRNA was performed in accordance with the manufacturer’s protocol. Relative expression levels were determined using the ΔΔCq method and normalized to GAPDH or RNU6 levels with Rio-Rad CFX Maestro software (CFX Maestro™ version 1.1; Bio-Rad). Data were expressed as the fold change in the treatment group relative to the control. The primer sequences are shown in Supplementary Table S1.

### 2.10. Western Blotting

Total protein was extracted using radioimmunoprecipitation (RIPA) buffer (Biosesang, Yongin, Republic of Korea) with a protease inhibitor (Roche Diagnostics, Mannheim, Germany). Total protein concentrations were measured using the Pierce™ BCA Protein Assay Kit (Thermo Fisher Scientific) in accordance with the manufacturer’s instructions. Protein samples with equal quantities were separated by 10% SDS–PAGE and transferred onto nitrocellulose membranes (Bio-Rad Laboratories, Hercules, CA, USA). The membranes were blocked with 5% BSA in Tris-buffered saline containing 0.1% Tween-20 (TBST, pH 7.6) at RT for 1 h. They were incubated with the appropriate primary antibody at RT for 1 h or at 4 °C overnight. Afterward, they were incubated with a secondary antibody at RT for 1 h. All primary and secondary antibodies were diluted to 1:1000 and 1:5000 in TBST, respectively. The primary and secondary antibodies used in this study are shown in Supplementary Table S2. The signals of the bands were visualized with an enhanced chemiluminescence method by using West Femto Maximum Sensitivity Substrate (Thermo Fisher Scientific) and the LuminoGraph II (WSE-6200; ATTO, Tokyo, Japan). Protein expression levels were quantified using ImageJ software [21].

### 2.11. Statistical Analysis

Data were obtained from at least three independent experiments, presented as the mean ± standard error of the mean (SEM), and analyzed using GraphPad Prism 8.0 (GraphPad Software, San Diego, CA, USA). Statistical analyses were performed using *t*-test or analysis of variance (ANOVA) followed by Bonferroni’s multiple comparison test; the exact *p* value (when *p* > 0.0001 but < 0.05) or the highest *p* value (*p* < 0.0001) were described.

## 3. Results

### 3.1. miR-21 Is Overexpressed in HBV-Associated HCC

**Figure 1:**
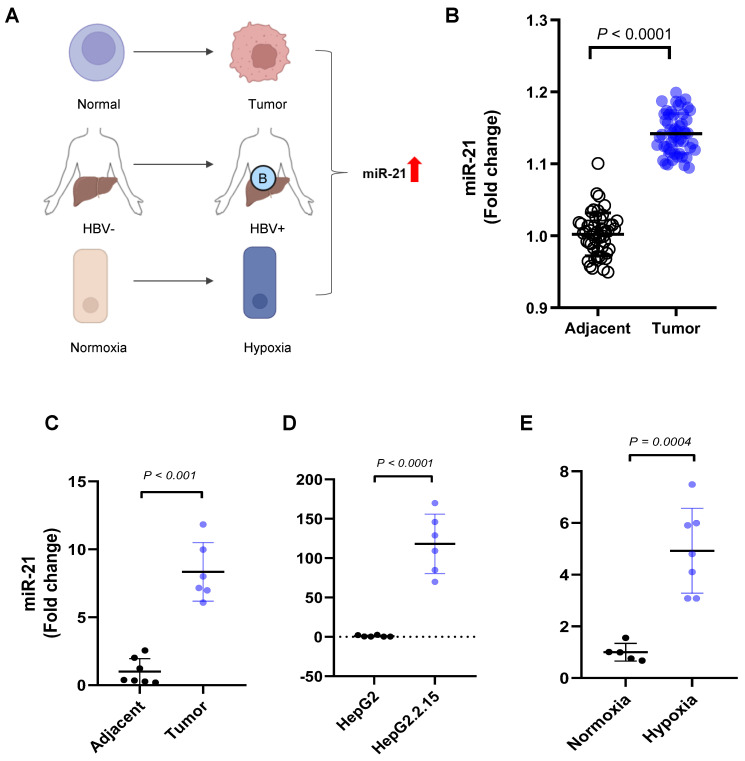
miR-21 expression in matched normal adjacent and tumor tissues and hepatitis B virus–related hepatocellular carcinoma cells. (**A**) Schematic illustration of miR-21 upregulation in HBV-positive HCC under hypoxic conditions. Red arrow highlight miR-21 upregulation. (**B**) miR-21 expression in tumor (blue) and adjacent non-tumor liver tissues (black) from the TCGA paired data set. (**C**) Relative miR-21 expression in human HBV-HCC tumor (blue) and normal tissues (black) measured by qRT-PCR. (**D**) Comparison of miR-21 expression levels in HepG2 (black) and HepG2.2.15 cells (blue). (**E**) Expression levels of miR-21 in HepG2.2.15 cells under normoxic (black) and hypoxic conditions (blue). Data are presented as mean ± SEM from at least three independent experiments (*N* = 6). Error bars represent SEM. Statistical analysis was performed using unpaired Student’s *t*-test. Data with *p* < 0.05 were considered significant.

The miR-21 expression was consistently elevated in HBV-associated HCC contexts (Figure 1A). In the TCGA dataset, the miR-21 expression was significantly higher in the tumor tissue than in the adjacent tissue (*p* < 0.0001; Figure 1B). To confirm this finding, we collected samples from patients who underwent hepatic resection surgery at Asan Medical Center and performed qRT–PCR analysis. The miR-21 expression level was increased in the HBV-HCC tissues compared with the adjacent noncancerous liver tissues (*p* < 0.0001; Figure 1B). Its basal expression in HepG2.2.15, the HBV DNA-integrated HepG2 cell line, was also increased compared with that of HepG2 (*p* < 0.0001; Figure 1C). In the following experiment that mimicked the HBV-activated condition of HBV-HCC, HepG2.2.15 cells were exposed to hypoxic conditions. The miR-21 expression was increased in HBV-HCC cells under a hypoxia-mimicking tumor microenvironment. The hypoxic marker miR-210 was upregulated during hypoxia (*p* = 0.0005; Figure 1D), and the relative miR-21 expression level was significantly increased (*p* = 0.0004; Figure 1E). Therefore, this elevated miR-21 expression might be associated with HBV-HCC progression and might further increase when exposed to oxidative stress.

### 3.2. SASH1 Is an miR-21 Target Gene That Decreases in HBV-HCC

**Figure 2:**
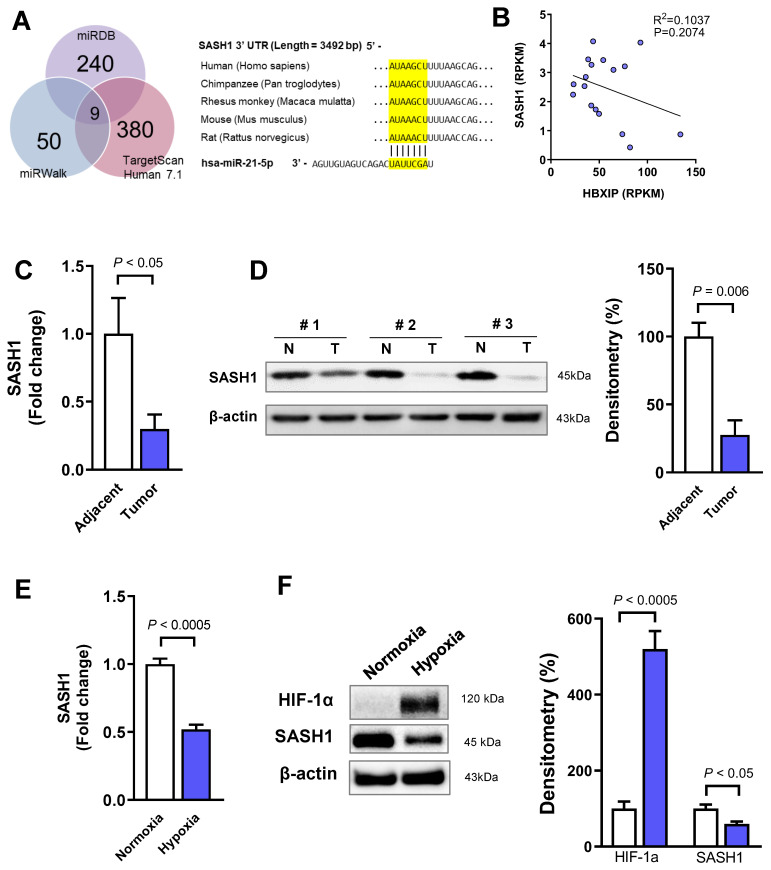
SASH1 is an miR-21 target gene downregulated in HCC under hypoxic conditions. (**A**) Venn diagram of the predicted miR-21 target genes from miRDB, miRWalk, and TargetScan Human 7.1 databases (yellow-highlighted sequences indicate the predicted miR-21 binding sites within the SASH1 3′-UTR). (**B**) Linear regression analysis of *HBXIP* and *SASH1* gene expression in the TCGA dataset. (**C**) Relative mRNA expression of the predicted target genes in HCC cells under hypoxic conditions. (**D**) Western blot analysis of SASH1 protein levels in paired adjacent normal (N) and tumor (T) tissues from patients with HCC. Densitometry was performed using ImageJ software. (**E**) mRNA expression of *SASH1* in HCC cells under normoxic and hypoxic conditions. (**F**) Western blot analysis of the hypoxia marker and SASH1 in HCC cells under hypoxia. Data are presented as mean ± SEM from at least three independent experiments (*N* = 5 or 6). Error bars represent SEM. For Western blots, representative images from at least three independent experiments are shown. Statistical analysis was performed using unpaired Student’s *t*-test. Data with *p* < 0.05 were considered significant. The uncropped Western blot figures can be found in Supplementary File S1.

To predict the target genes of miR-21, we searched for candidates by using the miRDB, miRWalk, and Target scan Human 7.1 databases. We selected nine genes as miR-21 targets (Figure 2A): *ALX4*, *BMPR2*, *MATN2*, *NFIB*, *PCBP2*, *SASH1*, *STAT3*, *TIMP3*, and *YOD1*. Among them, *SASH1* (SAM and SH3 domain containing 1) is known as a tumor suppressor. The TCGA dataset-based analyses revealed that the HBx protein-interacting-gene (HBxIP) expression was negatively correlated with *SASH1*, but the correlation was not significant (*p* = 0.2074; Figure 2B). The mRNA expression of *SASH1* was decreased in the tumor tissue compared with that in the adjacent normal tissue from patients with HBV-HCC (*p* < 0.05; Figure 2C). The SASH1 protein level was also downregulated in the tumor tissue (*p* < 0.005; Figure 2D). Subsequently, we exposed the HCC cells to hypoxia for 6 h to mimic the tumor microenvironment and extracted the total mRNA for qRT–PCR analysis. The mRNA level of *SASH1* was downregulated during hypoxia (*p* < 0.0005; Figure 2E). *SASH1* increased under hypoxic conditions, as confirmed by HIF-1α, a hypoxia marker (Figure 2F). These results suggested that *SASH1* was targeted and downregulated by miR-21 in HBV-HCC.

### 3.3. miR-21 Directly Binds to the 3′-UTR of SASH1

**Figure 3:**
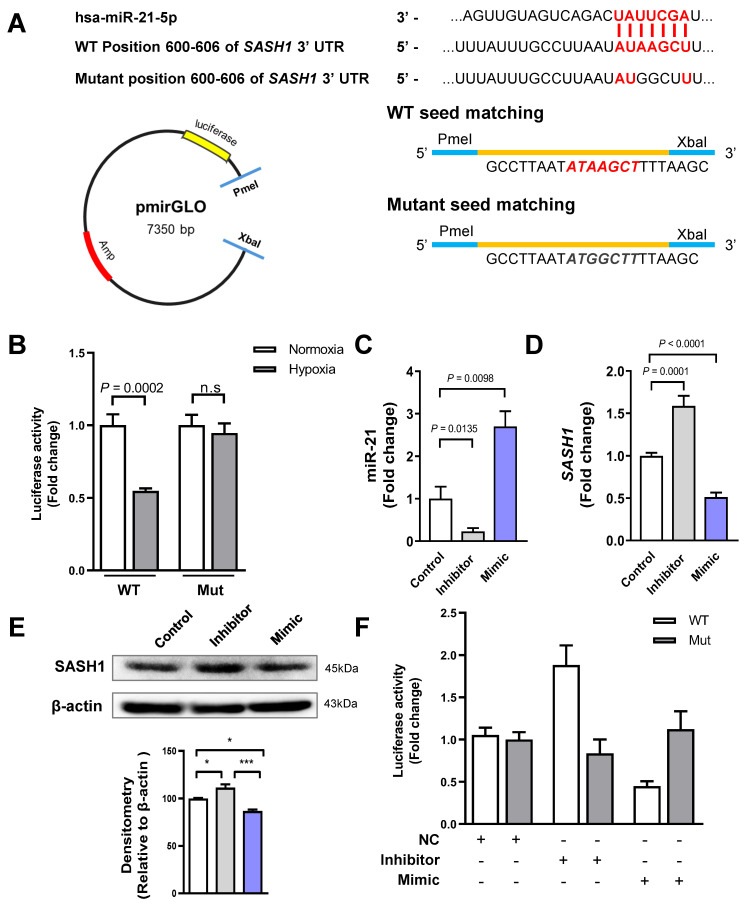
miR-21 directly targets the 3′-UTR of *SASH1* in HBV-HCC cells. (**A**) Schematic representation of the predicted miR-21 binding sites in the 3′-UTR of *SASH1* and vector map. (**B**) Luciferase reporter assay showing the interaction of miR-21 with the predicted binding sites in the 3′-UTR of *SASH1* under normoxic and hypoxic conditions. (**C**) qRT-PCR analysis of miR-21 and (**D**) *SASH1* expression in HCC cell lines transfected with miR-21 inhibitor or mimic. (**E**) Western blot analysis of SASH1 in the transfected HCC cells. Densitometry was performed using ImageJ software. (**F**) Luciferase activity in HCC cells co-transfected with recombinant vectors and miR-21 inhibitor, mimic, or negative control (NC). Data are presented as mean ± SEM from at least three independent experiments (*N* = 7). Error bars represent SEM. For Western blots, representative images from at least three independent experiments are shown. Statistical analysis was performed using unpaired Student’s *t*-test or one-way ANOVA followed by Bonferroni’s multiple comparison test. Data are shown as mean ± SD. n.s, not significant, * *p* < 0.05, *** *p* < 0.001 via one-way ANOVA. The uncropped Western blot figures can be found in Supplementary File S1.

To ensure that miR-21 directly interacted with *SASH1*, we designed a construct *SASH1* 3′-UTR with a WT or Mut miR-21 binding site. With these constructs, we then established a luciferase assay. Before cloning, we investigated *SASH1* 3′-UTR–binding miR-21 by using the Target Scan web-database and inserted the synthesized oligo into the pmirGLO vector (Figure 3A). We transfected the HBV-HCC cell lines with the recombinant vector for 6 h under hypoxic conditions. At the end of transfection, we measured the luciferase activity. The luciferase activity of WT 3′-UTR was decreased during hypoxia (*p* = 0.0002); conversely, the Mut form did not significantly change (Figure 3B). Next, we transfected the HCC cells with miR-21 inhibitor or mimic and performed qRT–PCR to detect gene expression. The inhibitor caused a decreased miR-21 and an increased *SASH1*, while the mimic-transfected HCC cells exhibited an increased miR-21 expression and a decreased *SASH1* (Figure 3C,D). Accordingly, the protein level of SASH1 increased with the miR-21 inhibitor and decreased when transfected with the miR-21 mimic (Figure 3E). When the HCC cells were co-transfected with *SASH1* 3′-UTR constructs and miR-21 inhibitor or mimic, the luciferase activity of the WT construct was increased by the miR-21 inhibitor (*p* < 0.0079) and decreased by the mimic (*p* = 0.0002). However, it did not change significantly in the HCC cells co-transfected with the Mut construct of either the inhibitor or mimic (Figure 3F). Therefore, *SASH1* could be directly targeted by miR-21.

### 3.4. Regulation of miR-21 and SASH1 Affects HBV-HCC Cell Viability

**Figure 4:**
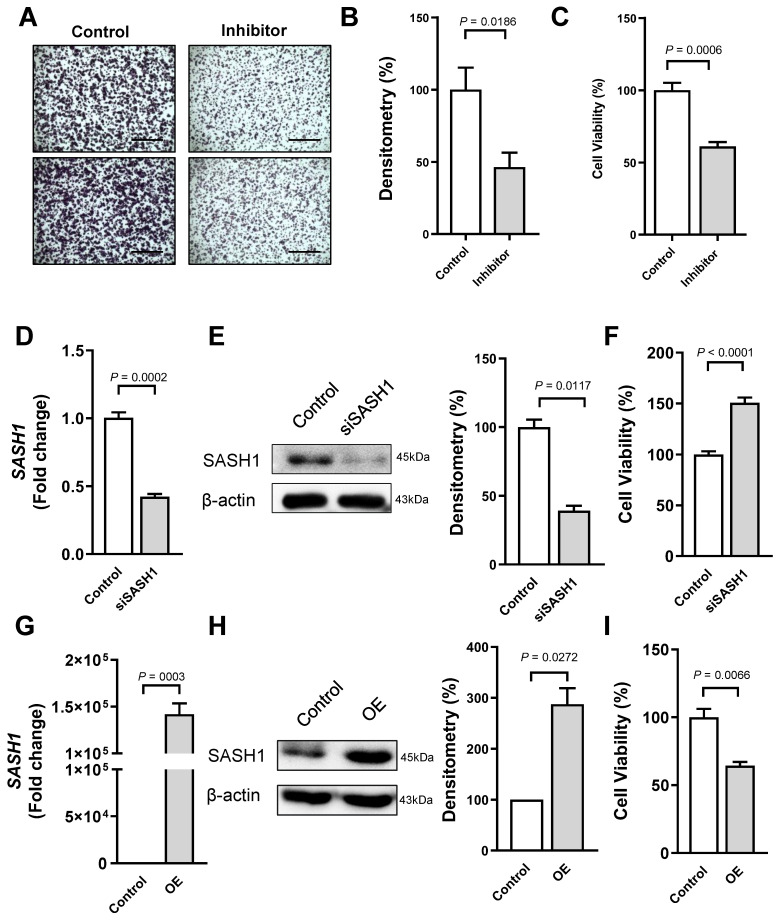
miR-21 inhibition suppresses HBV-HCC cell survival by regulating *SASH1*. (**A**) Representative images of crystal-violet-stained colonies formed by HCC cells transfected with the miR-21 inhibitor or negative control (NC). Scale bar: 1 mm. (**B**) Quantification of colony density measured using ImageJ software. (**C**) Cell viability of transfected HCC cells measured by MTT assay. (**D**) qRT-PCR analysis of *SASH1* expression in HCC cells transfected with siSASH1 or NC. (**E**) Western blot analysis of SASH1 in transfected HCC cells. Densitometry was performed using ImageJ software. (**F**) Cell viability of HCC cells transfected with siSASH1 or NC, measured by MTT assay. (**G**) qRT-PCR analysis of the mRNA expression of SASH1 in HCC cells transfected with the *SASH1* overexpression (OE) vector or empty vector control. (**H**) Western blot analysis of SASH1 protein levels in cells transfected with the OE vector or empty vector. Densitometry was performed using ImageJ software. (**I**) Viability of HCC cells transfected with the OE vector or empty vector, measured by MTT assay. Data are presented as mean ± SEM from at least three independent experiments (*N* = 3). Error bars represent SEM. For Western blots, representative images from three independent experiments are shown. Statistical analysis was performed using unpaired Student′s *t*-test. Data with *p* < 0.05 were considered significant. The uncropped Western blot figures can be found in Supplementary File S1.

The miR-21 expression was increased in HBV-HCC tissues and cells. The miR-21 inhibitor was transfected into HBV-HCC cells to perform a functional analysis. The colony formation assay revealed that the inhibitor attenuated the cell proliferation of HBV-HCC compared with that of the NC (Figure 4A). The density showed consistent results (Figure 4B). Meanwhile, the MTT assay showed that viability of HBV-HCC cells transfected with the inhibitor was significantly decreased compared with that of the control (*p* = 0.0007; Figure 4C).

miR-21 disrupted the *SASH1* expression (Figure 3). Alternating miR-21 overexpression, siSASH1 was transfected within HBV-HCC cells, and *SASH1* expression was detected. The SASH1 protein level was downregulated in siSASH1-transfected cells for 24 h (Figure 4D,E). However, the MTT assay showed that the viability of HBV-HCC cells transfected with siSASH1 was increased compared with that of the control (Figure 4F). Otherwise, HBV-HCC cells were transfected with the *SASH1* overexpression (OE) vector mimicking the miR-21 inhibitor. *SASH1* mRNA of the transfected cells was significantly upregulated compared with that of the control, but it increased until 12 h in a time-dependent manner (Figure 4G). The protein level of SASH1 also increased in the OE-vector-transfected cells (Figure 4H). Furthermore, the MTT assay revealed that OE vector transfection inhibited the viability of HCC cells (Figure 4I). These results demonstrated that the recovery of the *SASH1* expression inhibited by miR-21 could attenuate cancer cell proliferation in HBV-HCC.

### 3.5. miR-21 Inhibitor Enhances Sorafenib Efficacy in HBV-HCC Xenograft Model

**Figure 5:**
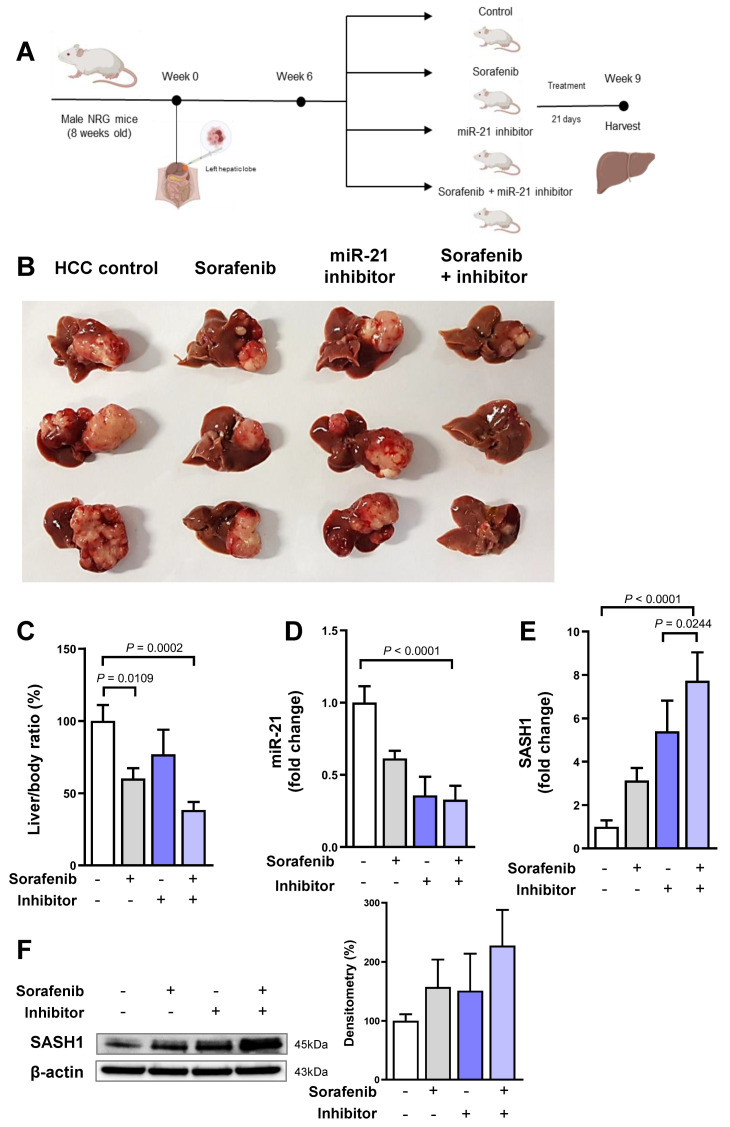
Enhanced effects of miR-21 inhibitor and sorafenib on tumor growth in an HBV-HCC xenograft mouse model. (**A**) Orthotopic HCC in the in vivo model. (**B**) Representative images of the extracted livers with tumor masses after 21 days of treatment. Scale bar: 1 cm. The liver image is representative of five or six independent experiments. (**C**) The liver/body weight ratio compared among the groups. The body weight of the mice was measured right before sacrifice. Data are presented as mean ± SEM from at least three independent experiments. (**D**) qRT–PCR analysis of miR-21 and (**E**) SASH1 expression levels in the liver tissues of the mouse model. Data are presented as mean ± SEM from at least three independent experiments (*N* = 6). (**F**) Western blot analysis of SASH1 in the mouse liver. The image shown is representative of three independent experiments. Statistical analysis was performed using unpaired Student′s *t*-test, and data with *p* < 0.05 were considered significant. The uncropped Western blot figures can be found in Supplementary File S1.

To investigate the in vivo combined effects of sorafenib and miR-21 inhibitor, we established an orthotopic xenograft HBV-HCC model with NRG mice (Figure 5A). At the end of the treatment, the size of the tumor mass of the mice orally injected with sorafenib was reduced; conversely, it did not significantly decrease in the mice treated with the miR-21 inhibitor. However, the tumor size decreased the most in the mice treated with both sorafenib and miR-21 inhibitor (sorafenib + inhibitor; Figure 5B). Although the liver/body ratio did not differ between the inhibitor-treated mice and the control, it decreased in the sorafenib-treated mice; the lowest ratio was detected in the mice treated with sorafenib + inhibitor (Figure 5C). The combination treatment significantly reduced the liver/body ratio compared with the single treatment. As we performed qRT–PCR with the RNA extracted from mouse liver, the relative miR-21 expression level was downregulated in the other groups compared with that in the control (Figure 5D); however, the mRNA expression of *SASH1* significantly increased (Figure 5E). Western blot analysis showed that the *SASH1* expression was higher in the sorafenib or miR-21 inhibitor treatment group, and SASH1 protein expression increased in both treatment groups (Figure 5F).

### 3.6. miR-21 Inhibition Enhances Sorafenib-Induced Apoptosis Through SASH1 Upregulation

**Figure 6:**
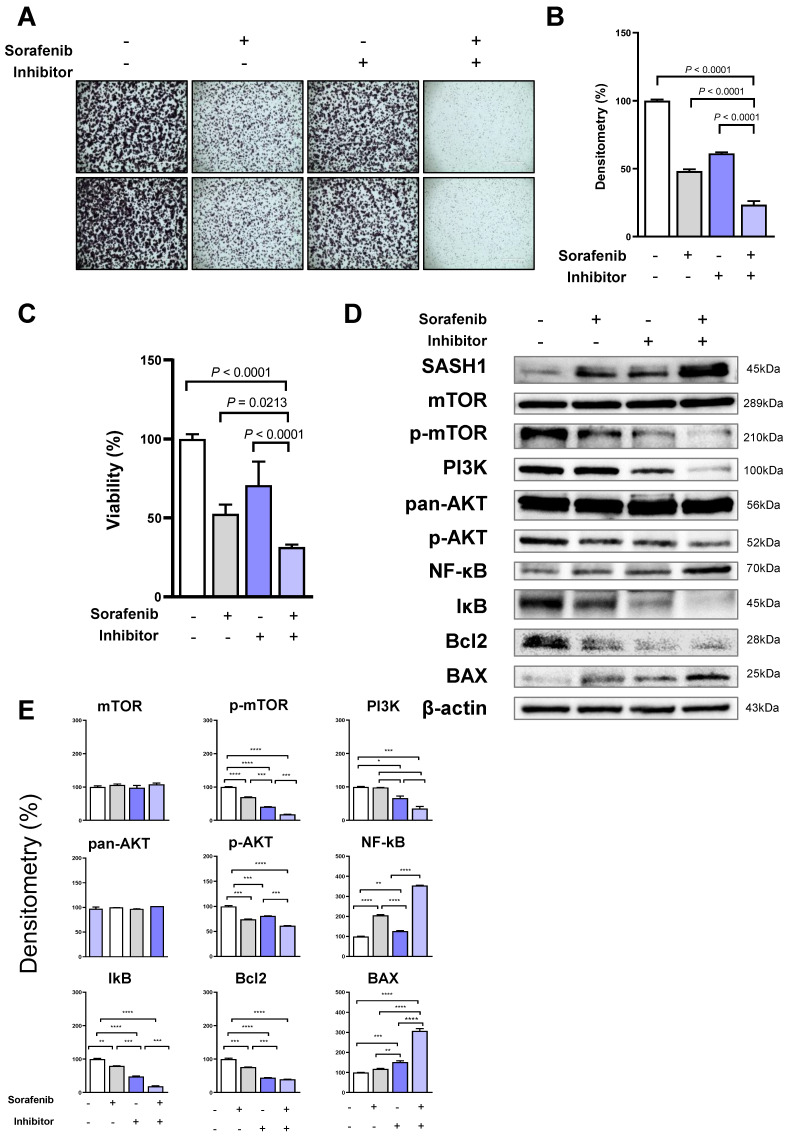
Enhanced effects of miR-21 inhibitor and sorafenib on HBV-HCC cell proliferation and apoptosis in vitro. (**A**) Crystal violet staining of HCC cell colonies after the treatment with sorafenib, miR-21 inhibitor, or their combination. Scale bar: 1 mm. (**B**) Quantification of colony density measured using ImageJ software. (**C**) Cell viability measured by MTT assay after various treatments. (**D**) Western blot analysis of apoptosis-related proteins in the treated HCC cells. Data are presented as mean ± SEM from at least three independent experiments. For colony formation and Western blot assays, representative images from three independent experiments are shown. Statistical analysis was performed using one-way ANOVA followed by Tukey’s post hoc test, and data with *p* < 0.05 were considered significant. (**E**) Densitometric quantification of Western blot results shown in panel (**D**). Protein expression levels were normalized to β-actin and expressed as percentages relative to the untreated control group. Data represent mean ± SD of independent experiments. Statistical significance is indicated * *p* < 0.05, ** *p* < 0.01, *** *p* < 0.001, **** *p* < 0.0001. The uncropped Western blot figures can be found in Supplementary File S1.

The combined effect was confirmed in vitro. HBV-HCC cells were treated with sorafenib, miR-21 inhibitor, or both. Crystal violet staining revealed that sorafenib and inhibitor attenuated the colony formation of HCC cells; however, the combined treatment most effectively inhibited colony formation (Figure 6A). It demonstrated the same densitometry results (Figure 6B). In addition, MTT assay was performed to detect cell viability. The results showed that cell viability was decreased in the cells treated with sorafenib and miR-21 inhibitor. Consistent with previous in vivo results, the combination treatment most effectively abolished the proliferation of HBV-HCC cells compared with each treatment alone (Figure 6C). Western blot analysis was performed to determine the mechanism. Interestingly, the combined treatment downregulated the levels of phospho-mTOR, PI3K, and phospho-AKT, while it increased the protein levels of mTOR and pan-AKT. NF-κB downstream of the mTOR/PI3K/AKT pathway was upregulated after the combination treatment; however, IκB, which inhibits NF-κB, decreased. Additionally, the combined treatment decreased the Bcl2 protein expression level but increased the BAX pro-apoptotic protein expression level (Figure 6D,E). These results demonstrated that miR-21 inhibition enhanced sorafenib efficacy in HBV-HCC by restoring the SASH1 expression. In summary, the combination treatment of miR-21 inhibitor and sorafenib further enhanced the suppression of the tumor growth and increased apoptosis in our HBV-HCC xenograft model. Therefore, it showed a potential therapeutic strategy for overcoming sorafenib resistance in HBV-associated liver cancer.

## 4. Discussion

This study identified miR-21 as a critical driver of HBV-related hepatocellular carcinoma and elucidated a previously unrecognized miR21/SASH1 regulatory axis that modulates sorafenib response by using HepG2.2.15, an HBV-infected HCC cell subtype, to establish a model mimicking HBV-positive HCC. Our data showed that the miR-21 expression significantly increased in HCC tissues and cell lines, particularly in HBV-positive samples. This finding aligned with previous studies demonstrating the aberrant expression of miRNAs in tumorigenesis [22,23,24]. Notably, miR-21 overexpression has been widely associated with poor prognosis and decreased survival outcomes in multiple cancer types [11,12,13,14,15]. Notably, miR-21 overexpression has been widely associated with poor prognosis and decreased survival outcomes in multiple HCC cancer types [11,12].

We observed that the miR-21 expression was negatively correlated with the SASH1 expression in HCC tissues and cell lines. SASH1, a known tumor suppressor gene, regulates cell survival, proliferation, and migration. SASH1 is also described as a scaffold-type protein that modulates oncogenic signaling pathways, in addition to its tumor-suppressive role. Previous studies demonstrated that the loss of SASH1 protein enhances pro-survival signaling and tumor progression [25,26,27,28]. Given that the persistent activation of the PI3K/AKT/mTOR pathway is a well-known mechanism of sorafenib resistance in HCC [6,7], restoring SASH1 protein may attenuate compensatory survival signaling and lower the apoptotic threshold in response to sorafenib treatment. Furthermore, our luciferase reporter assay confirmed that miR-21 directly targets the 3′-UTR of SASH1, suggesting a novel regulatory mechanism in tumor progression.

We further showed that hypoxic conditions markedly enhanced the miR-21 expression. HBV infection generates excessive reactive oxygen species (ROS) and creates a hypoxic microenvironment. In this study, we used a hypoxic culture condition to mimic this pathophysiological state. Interestingly, hypoxia induced higher miR-21 expression levels, implying that HBV-induced ROS might contribute to the enhanced miR-21 expression. This finding provided a potential link between HBV infection, oxidative stress, and miRNA dysregulation in HCC progression [5,29].

We found that miR-21 upregulation suppressed the SASH1 expression and limited sorafenib-induced apoptotic cell death, whereas miR-21 inhibition restored SASH1 levels and improved the cellular response to sorafenib. SASH1 demonstrated the ability to promote tumor cell death induced by sorafenib-triggered apoptosis independent of the miR-21 expression. The significant reduction in this cell death effect in SASH1-deficient conditions suggested a potential interaction between HBx and SASH1. Our experiments with miR-21 and SASH1 overexpression/knockdown revealed an inverse relationship between SASH1 and HBx expression. Therefore, SASH1 might suppress HBx gene expression when adequately expressed. This finding provided insights into the complex interplay between viral factors and host tumor suppressors in HBV-HCC.

Our findings suggested that miR-21 regulated sorafenib sensitivity in HBV-associated HCC by suppressing SASH1. The increased miR-21 expression under HBV-related or hypoxic conditions reduced SASH1 levels, resulting in the sustained activation of HBx-associated PI3K/AKT/mTOR survival signaling and decreased susceptibility to sorafenib-induced apoptosis. Conversely, miR-21 inhibition restored SASH1 expression, weakened pro-survival signaling, and enhanced apoptotic responses following sorafenib treatment. These results indicated that miR-21/SASH1 axis influenced therapeutic responsiveness and contributed to sorafenib resistance in HBV-positive HCC. Nevertheless, our study has limitations. First, we were unable to directly compare miR-21 expression levels between HBV-positive and HBV-negative HCC; this comparison could provide further insights into the virus-specific effects on miRNA dysregulation. Second, our clinical cohort exhibited a gender imbalance, and only male mice were used in the animal experiments. Given the higher incidence of liver cancer in males [1], future studies should investigate potential sex-specific differences in miR-21 expression and its therapeutic targeting in HCC. Third, although our findings suggested a functional association between SASH1 and HBx-related signaling, we did not perform direct biochemical assays such as a immunoprecipitation to confirm a physical interaction. In addition, further studies should determine whether the modulation of the miR-21/SASH1 axis might enhance the therapeutic efficacy of other systemic agents such as lenvatinib used in HCC treatment.

In conclusion, our study provided evidence describing a miR-21/SASH1 regulatory axis in HBV-positive HCC that might contribute to sorafenib resistance. These findings not only enhanced our understanding of HBV-related hepatocarcinogenesis but also suggested new therapeutic strategies. Future research should clarify the downstream pathways linking SASH1 to HBx regulation and evaluate the translational applicability of miR-21-targeted therapies, including combination strategies with existing systemic treatments, for patients in diverse HCC subgroups.

## 5. Conclusions

In summary, this study demonstrated that miR-21 was upregulated in HBV-associated hepatocellular carcinoma and contributed to sorafenib resistance by suppressing SASH1. We showed that miR-21 directly targeted the 3′-UTR of SASH1, and the reduced SASH1 expression was associated with enhanced HBx-related oncogenic signaling and decreased apoptotic responses to sorafenib. These findings suggested that miR-21-SASH1 axis modulated therapeutic responses in HBV-positive HCC.

Pharmacologic inhibition of miR-21 restored SASH1 expression and enhanced the anti-tumor effects of sorafenib in both in vitro and in vivo models. The combination treatment further reduced tumor growth and increased apoptotic signaling.

Our findings provided mechanistic insights into the interaction between oncogenic factors and host tumor suppressor pathways in HBV-related HCC. Further studies should determine the clinical relevance for miR-21 targeting and its potential role as an adjunct to existing systemic therapies.

## Figures and Tables

**Table 1:** Clinical characteristics of patients with hepatocellular carcinoma.

No.	Sex	Age (Years)	Blood Type	Diagnosis	HBV	HCV	AFP (ng/mL)	PIVKA-II (mAU/mL)
1	M	61	AB+	Hepatocellular carcinoma	Yes	No	4.8	45
2	M	62	AB+	Liver cirrhosis due to hepatitis B	Yes	No	1.7	61
3	F	51	AB+	Hepatocellular carcinoma	Yes	No	98.1	47
4	M	52	A+	Advanced HCC, chronic viral hepatitis B with delta-agent	Yes	No	21,848	69,296
5	M	56	B+	Hepatocellular carcinoma, chronic viral hepatitis B	Yes	No	58.7	262
6	M	69	A+	Hepatocellular carcinoma	Yes	No	3.6	22
7	M	54	A+	Hepatocellular carcinoma (HBV LC r/o HCC)	Yes	No	6.6	110
8	M	57	B+	Hepatocellular carcinoma (HBV LC HCC)	Yes	No	4.5	84

HBV: Hepatitis B virus; HCV: Hepatitis C virus; AFP: Alpha-fetoprotein (normal range, <7.5 ng/mL); PIVKA-II: Protein induced by vitamin K absence or antagonist-II (normal range, <40 mAU/mL); LC: Liver cirrhosis; r/o: Rule out; HCC: Hepatocellular carcinoma.

## Data Availability

Data are available from the corresponding author upon reasonable request. The TCGA dataset analyzed in this study is publicly available from the Broad TCGA GDAC Firehose (http://gdac.broadinstitute.org/). Additional data may be requested from the authors.

## Supplementary Materials

The following supporting information can be downloaded at: https://www.mdpi.com/article/10.3390/cancers18061038/s1, File S1: The uncropped Western blot figures; Table S1: Primer information; Table S2: Antibody list; Table S3: siRNA sequences and plasmid construction details for SASH1 overexpression.
