# Supplementary material for: Therapeutic Targeting of miR-21 Restores SASH1 and Sensitizes HBV-HCC to Sorafenib
Source: Cancers (Basel). 2026 Mar 23;18(6):1038. doi: 10.3390/cancers18061038 (PMC13025902; doi:10.3390/cancers18061038)
Supplement: Supplementary file 1 [file cancers-18-01038-s001.zip › cancers-4176596-supplementary.pdf]

Supplementary Table S1. Primer information.

Gene	Sense or Antisense	Sequence (5'-3')
		mRNA
GAPDH	Sense<br>Antisense	GAGTCAACGGATTTGGTCGT<br>TTGATTTTGGAGGGATCTCG
SASH1	Sense<br>Antisense	AGATTTGGACACCTTTAAGC<br>CTGTCATACTCTTGTAACAGC
		miRNA
RNU6	Sequence not provided	Hs_RNU6-2_11 miScript Primer Assay (Cat no. MS00033740; Qiagen)
miR-21	Sequence not provided	Hs_miR-21_2 miScript Primer Assay (Cat **n**o. MS00009079; Qiagen)
miR-210	Sequence not provided	Hs_miR-210-5p_1 miScript Primer Assay (Cat no. MS00045836; Qiagen)

Supplementary Table S2. Antibody list.

Protein	Manufacturer	Catalog No.
SASH1	Santa Cruz	sc-517001
HIF-1α	BD	610958
mTOR	Abcam	ab2732
p-mTOR (phospho-mTOR)	Santa Cruz	sc-293133
PI3K	Abcam	ab40776
pan-AKT	Abcam	ab8805
p-AKT (phospho-AKT)	Santa Cruz	sc-514032
NF-kB	Santa Cruz	sc-8008
IkB	Santa Cruz	sc-1643
Bcl2	Santa Cruz	sc-7382
BAX	Santa Cruz	sc-7480
β-actin	Sigma Aldrich	A3854
mouse-IgGκ BP-HRP	Santa Cruz	sc-516102
goat anti-rabbit IgG-HRP	Santa Cruz	sc-2004

Supplementary Table S3. siRNA sequences and plasmid construction details for SASH1 overexpression.

Gene		Sequence (5′–3′)
SASH1 (siRNA)		CAGGUUUGUUCUAGUCAAA
SASH (OE)	Vector backbone	pcDNA3.0
	5′ Restriction enzyme	HindIII
	3′ Restriction enzyme	Xbal
	Insert gene	SASH1 (516 bp)
	Coding sequence	AGCGTTCAATGCATTTCACGTGCTGTGGTGGAT<br>GTGGGTGCTGTAGACAGGCTTCTTCTCTTCCTG<br>CTCTCAAAATACCTCGGCTTGACATTTGGACAG<br>ATCCTGTCATTGTTTAAGCTGAGCAAAAAACCA<br>CACAAAAGTTGTGTAAGAGATGAGATAACAAA<br>GGAGCGAGAGAAATCTCATGTGAATTTCCAAGT<br>TTTAATTCGTTCTCCATGAAGGATTTTCATTTCA<br>GTGAAAGTCGCAGCAGAAGAGGGAACTTTCTG<br>GAGTTTTTGAGAATGCCAAACCACATTTTTATCA<br>CACTTCTTTGGAAATCAATGCCTTTGCATAGAA<br>AATCAAATTCAGGGACCACAAAGAATTTTCAGT<br>GGGAATGTCTAGTCTGAGGGGTCTGAGGTTGTT<br>TTTACTTTATTGTGTTGTTTAAATATTTTAAAAAT<br>ATCTTTAGCGTTTGGTCTTTTTTTTTCTGTAAA<br>CATTTAATTTGGTCTGAGAAAAGCTGAATGTTT<br>GGGTGTGACGTTTGACTG
