# Supplementary material for: Therapeutic Targeting of miR-21 Restores SASH1 and Sensitizes HBV-HCC to Sorafenib
Source: Cancers (Basel). 2026 Mar 23;18(6):1038. doi: 10.3390/cancers18061038 (PMC13025902; doi:10.3390/cancers18061038)

Fig 2

D

SASH1

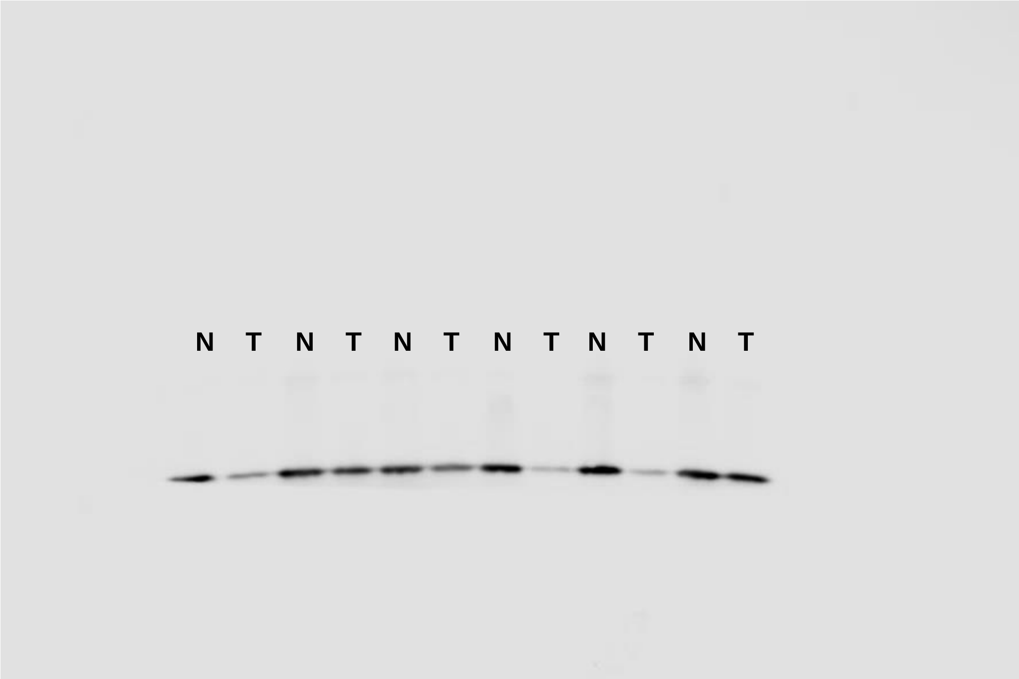

β-actin

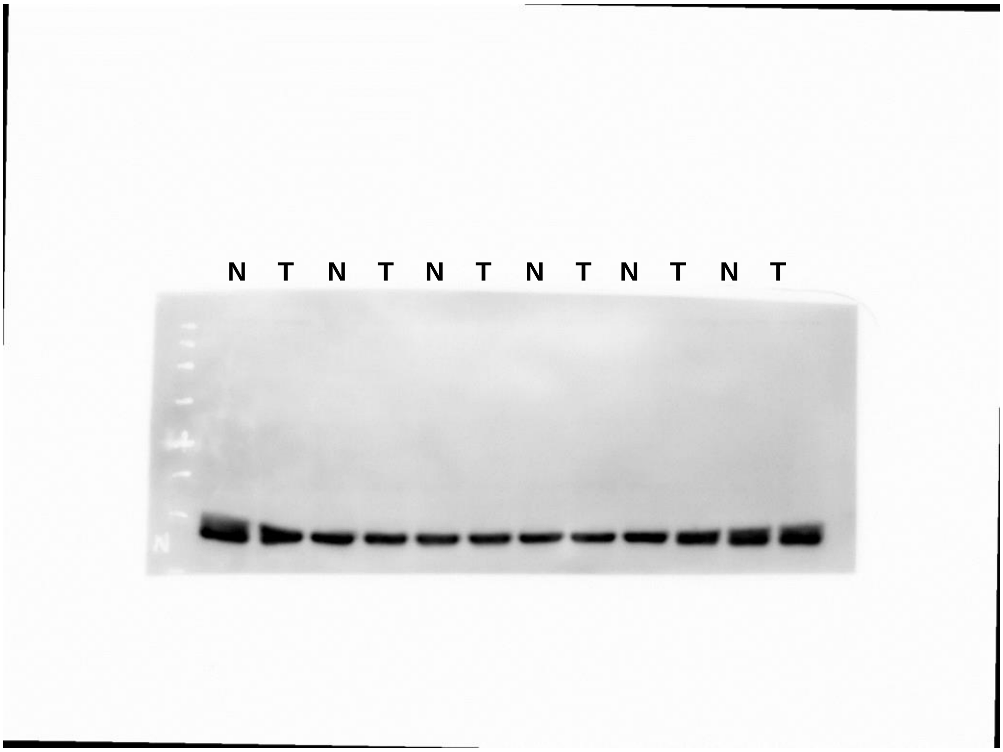

Fig 2

F

HIF-1α

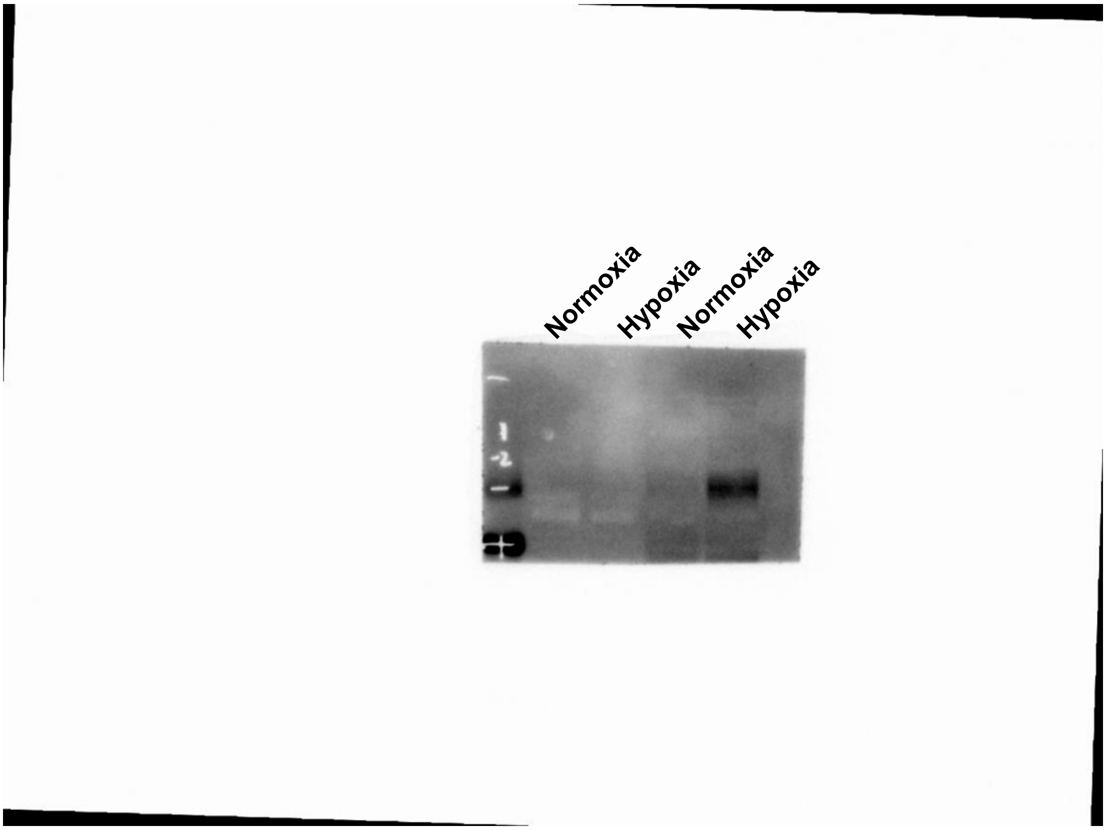

SASH1

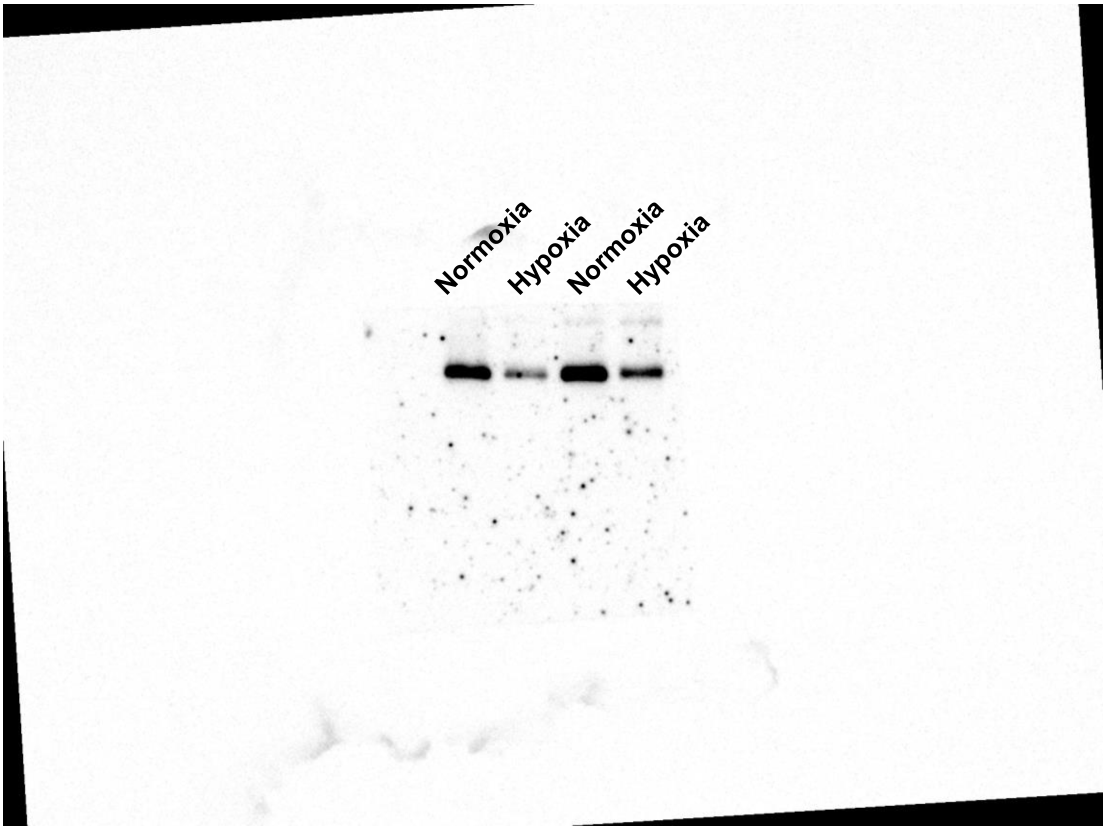

Fig 2

F

β-actin

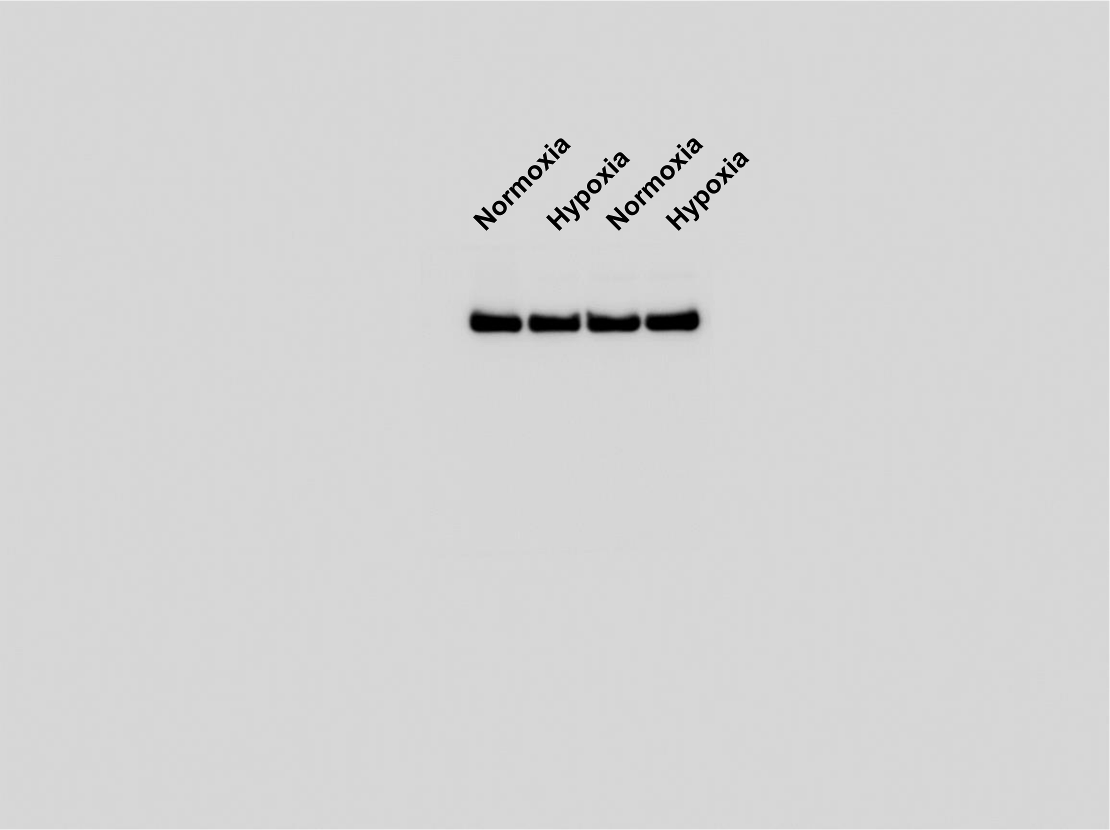

Fig 3

E

SASH1

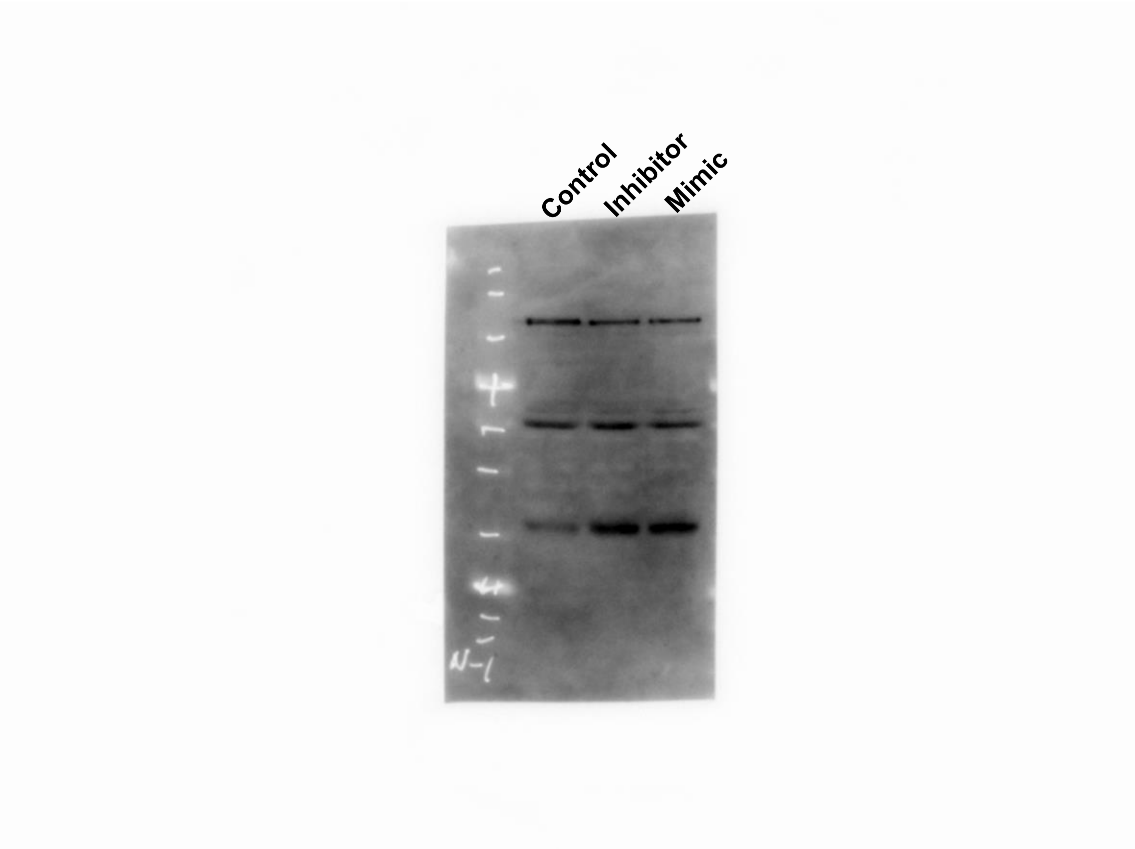

β-actin

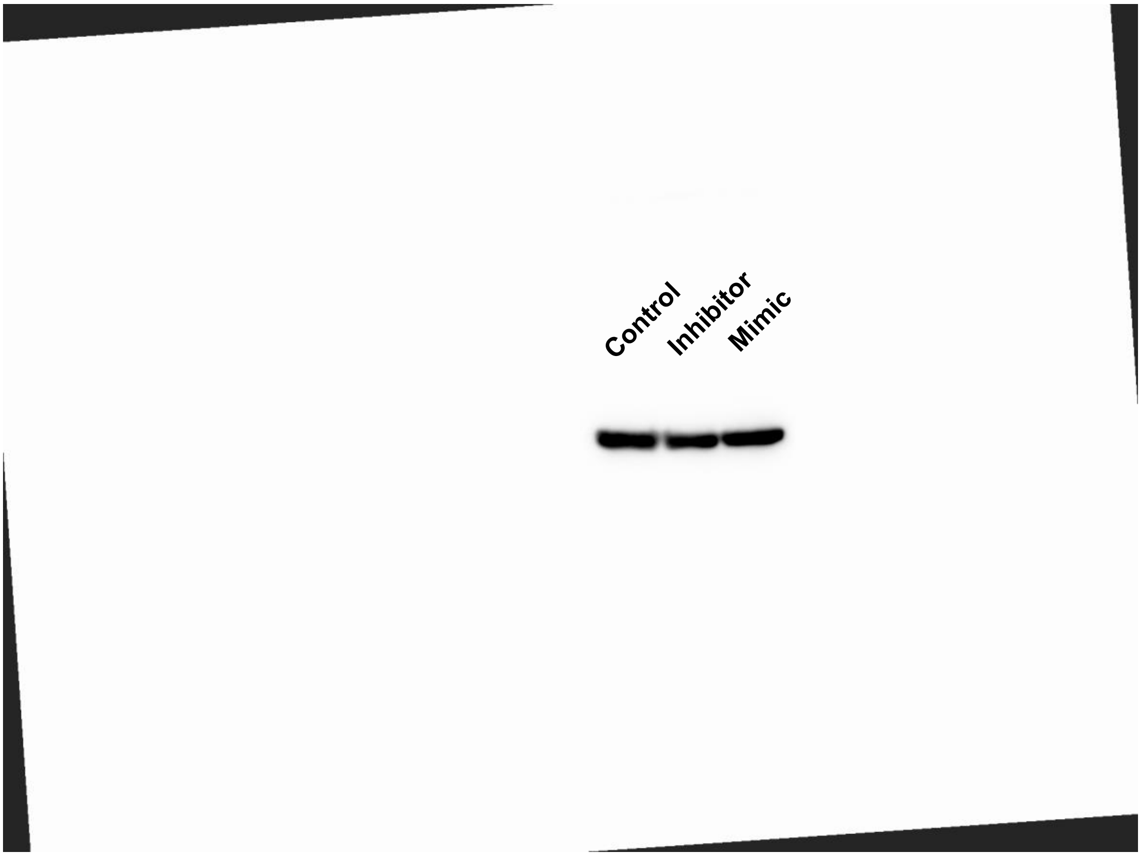

Fig 4

B

SASH1

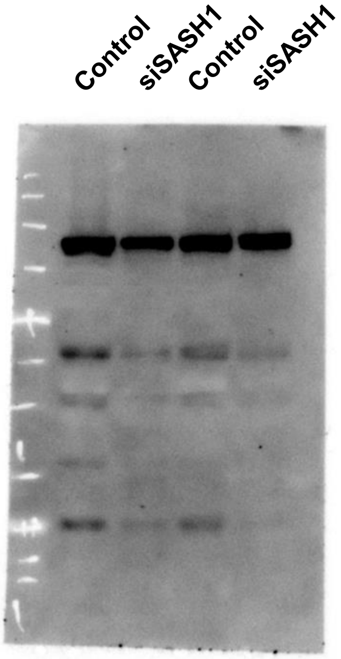

β-actin

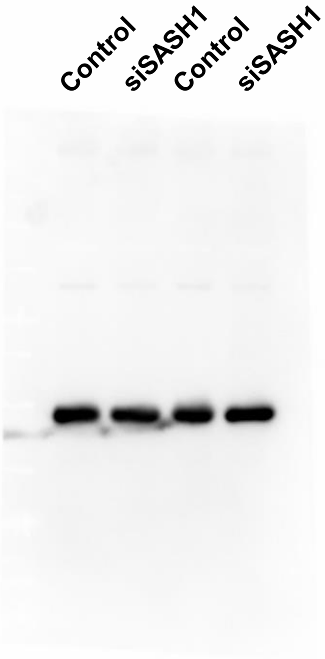

Fig 4

H

SASH1

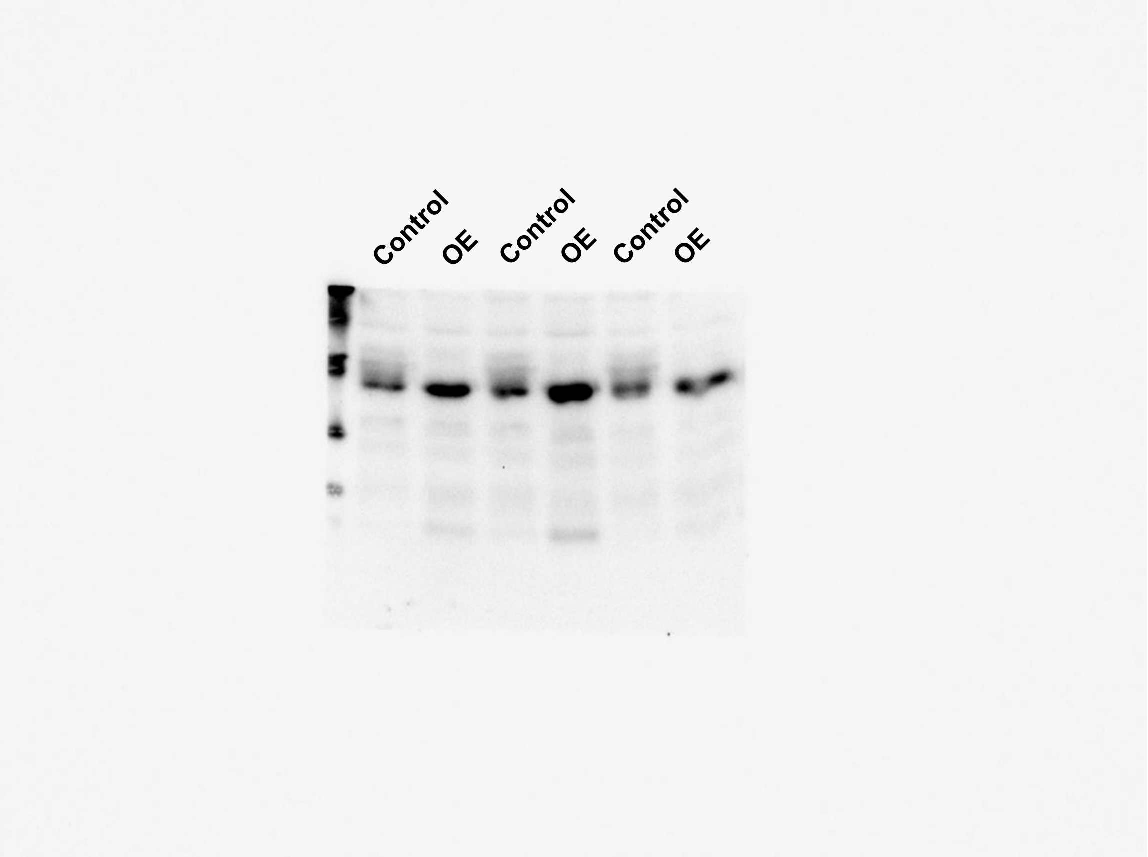

β-actin

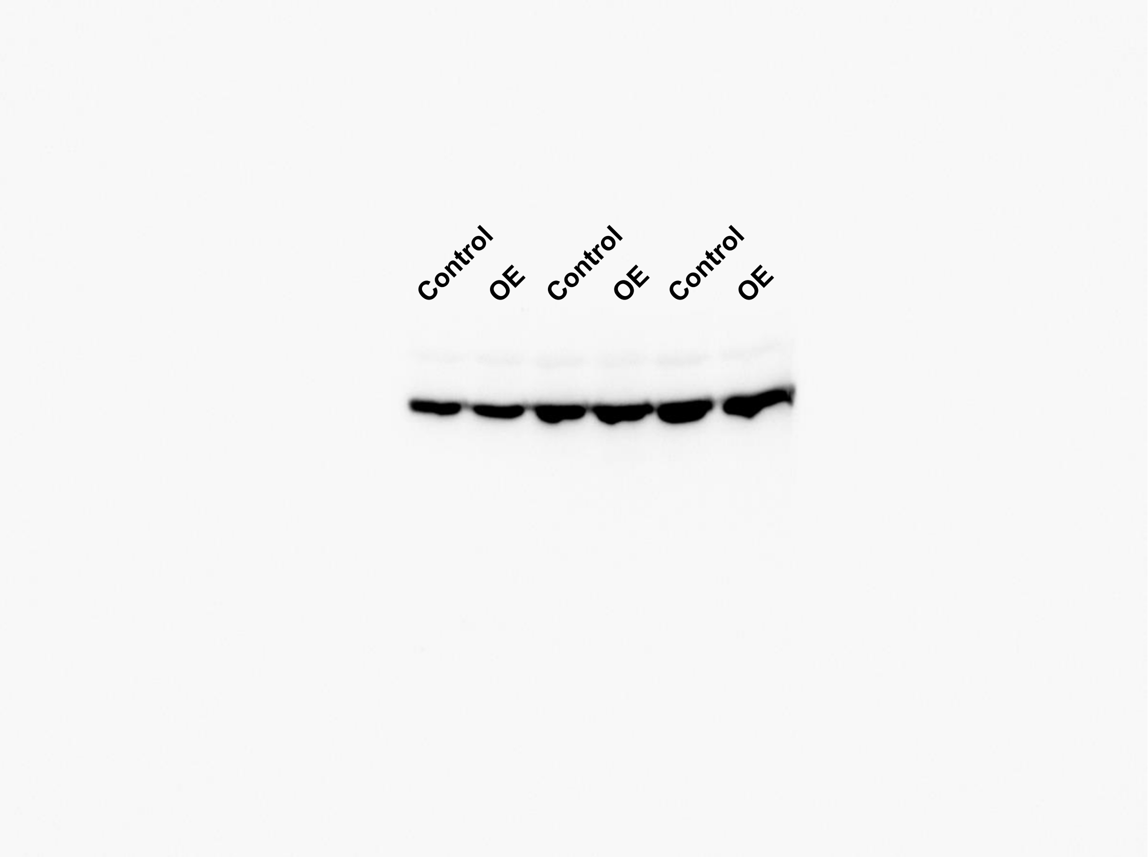

Fig 5

E

SASH1

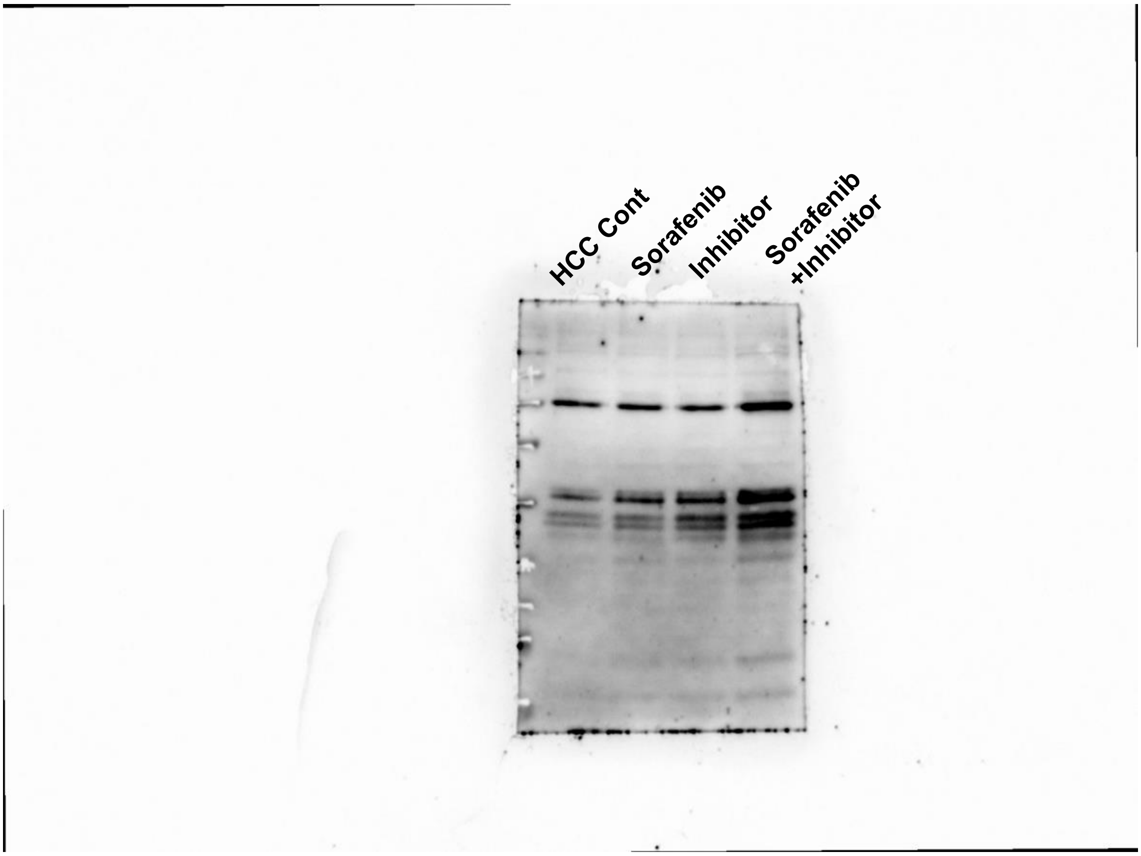

β-actin

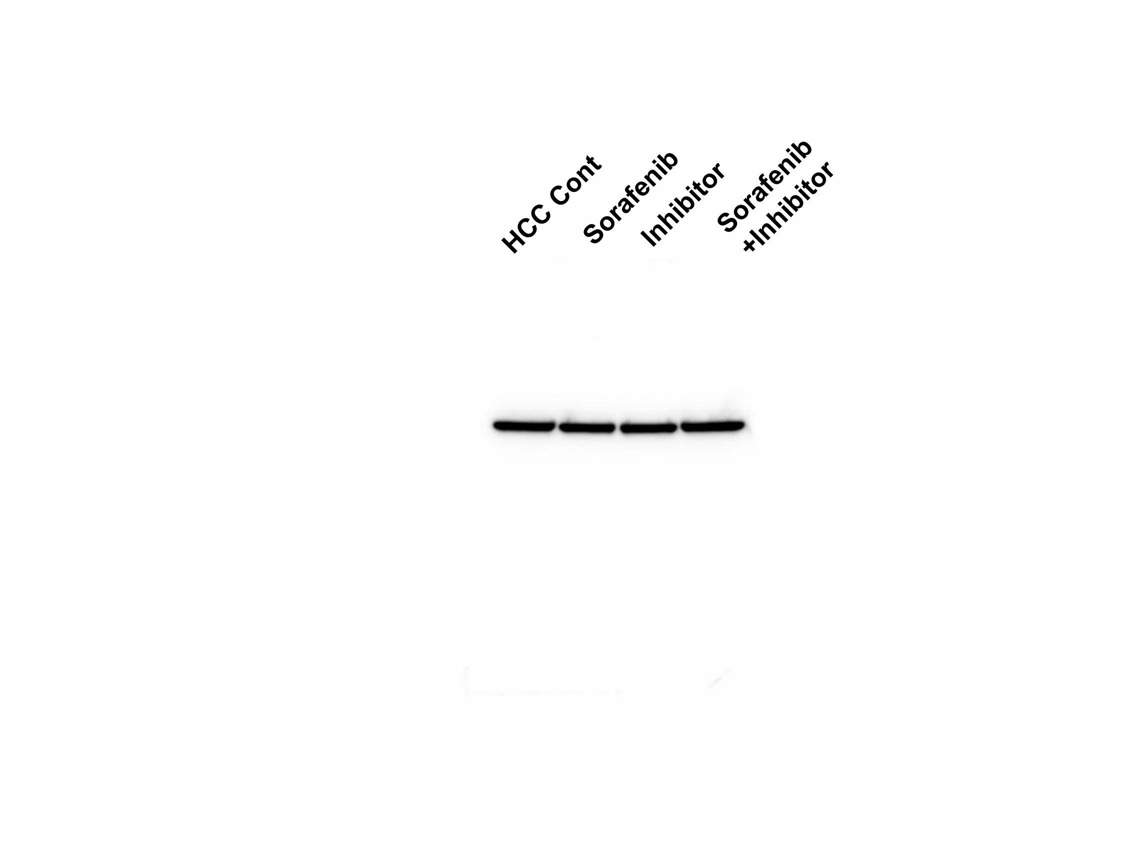

Fig 6

D

SASH1

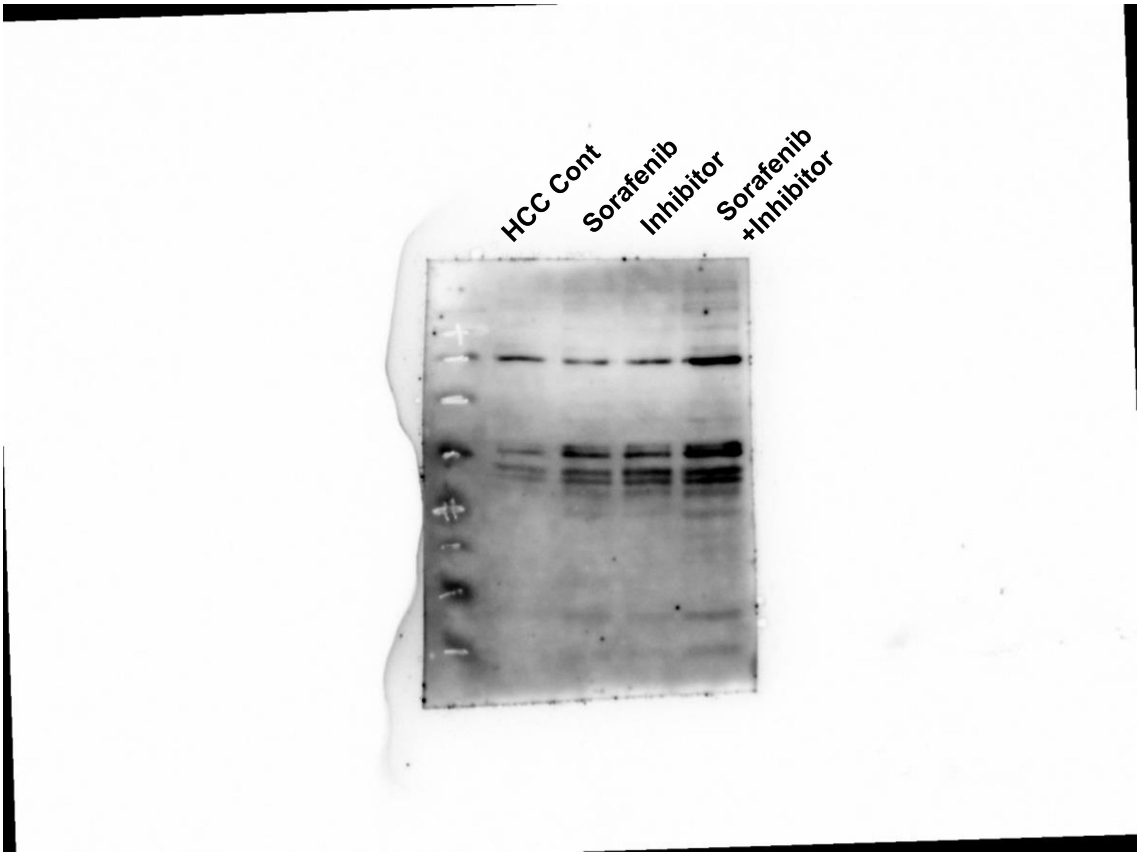

PI3K

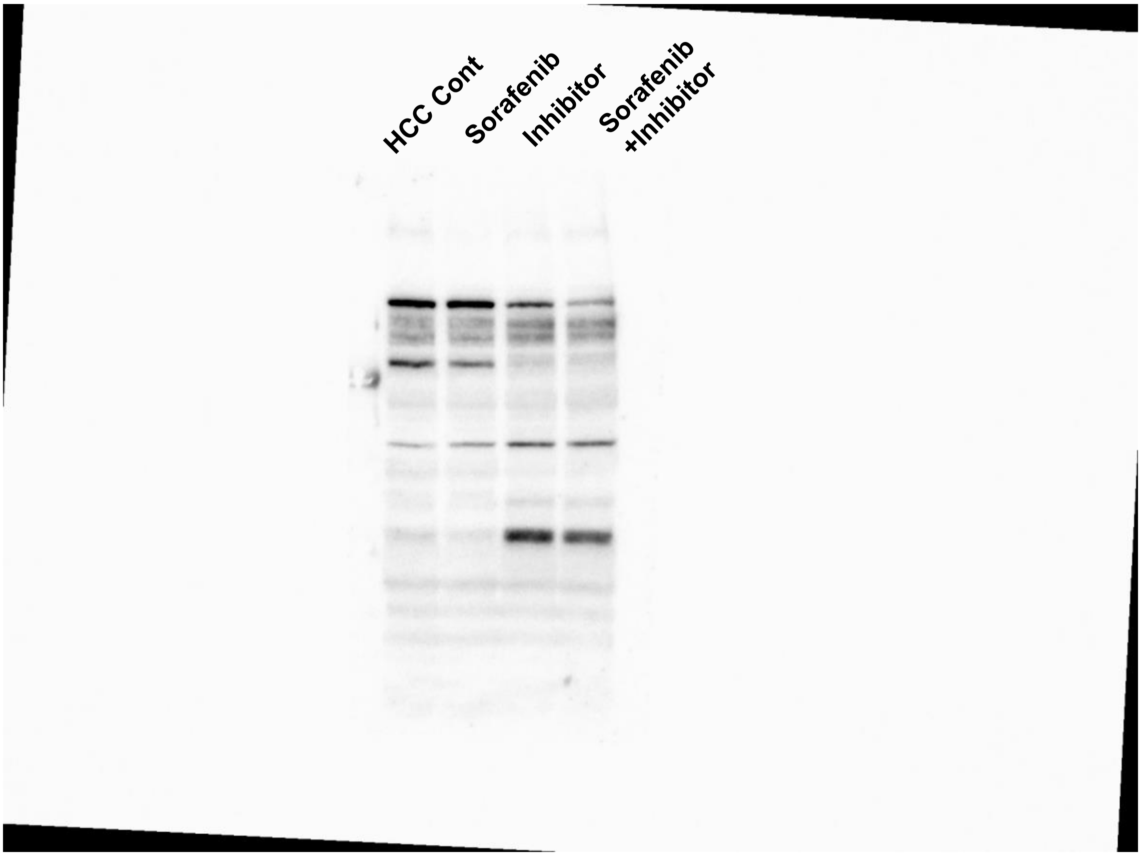

Fig 6

D

mTOR

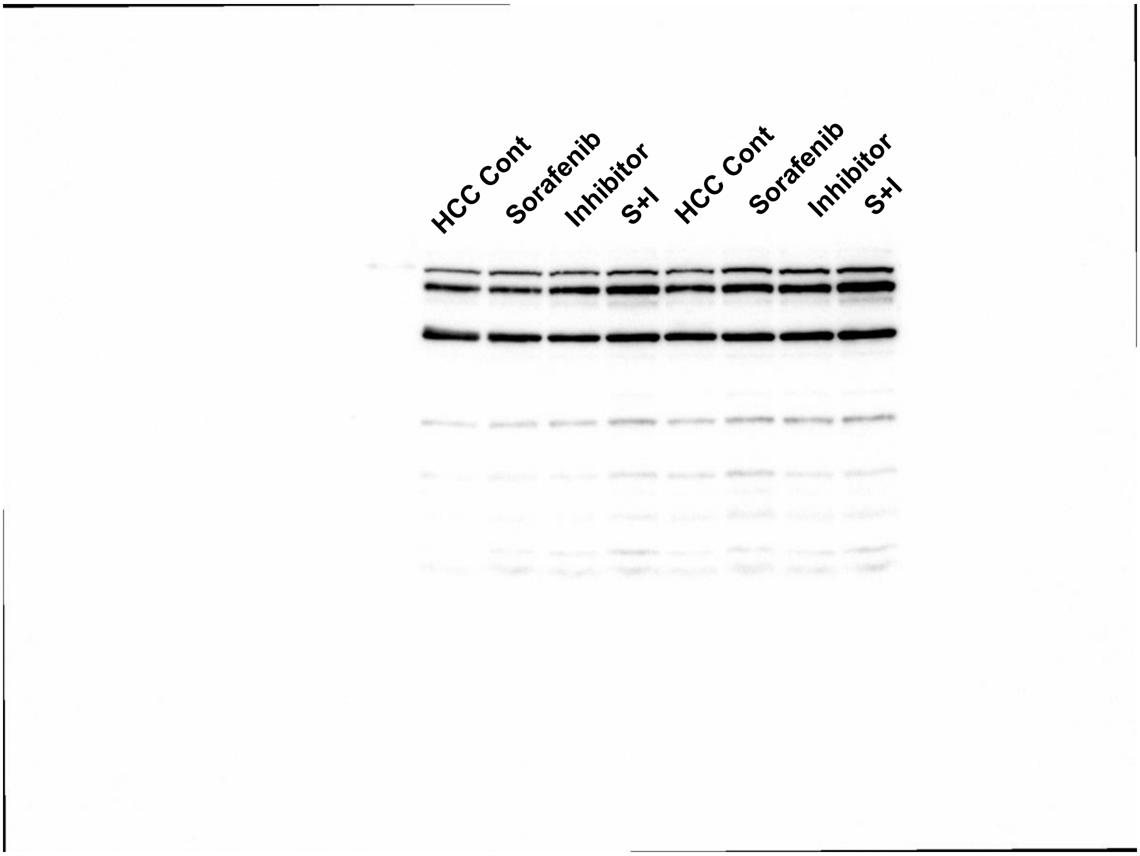

p-mTOR

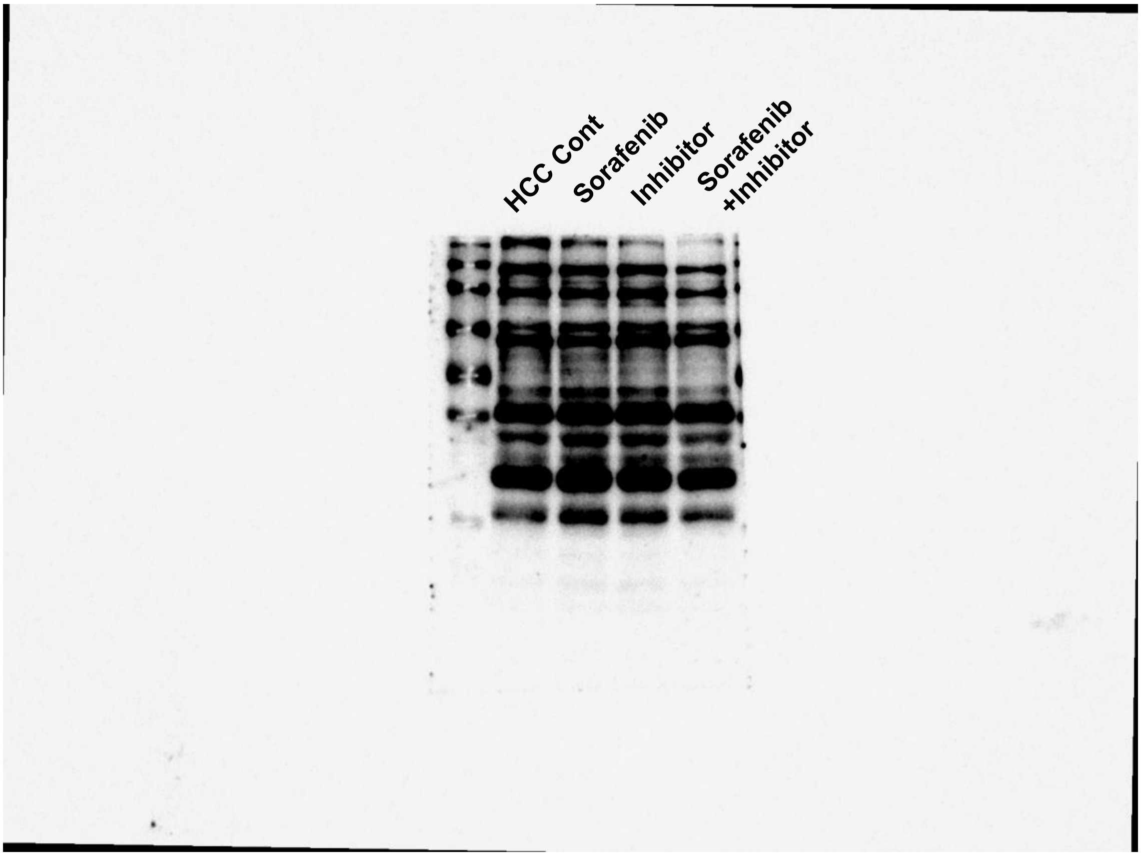

Fig 6

D

pan-AKT

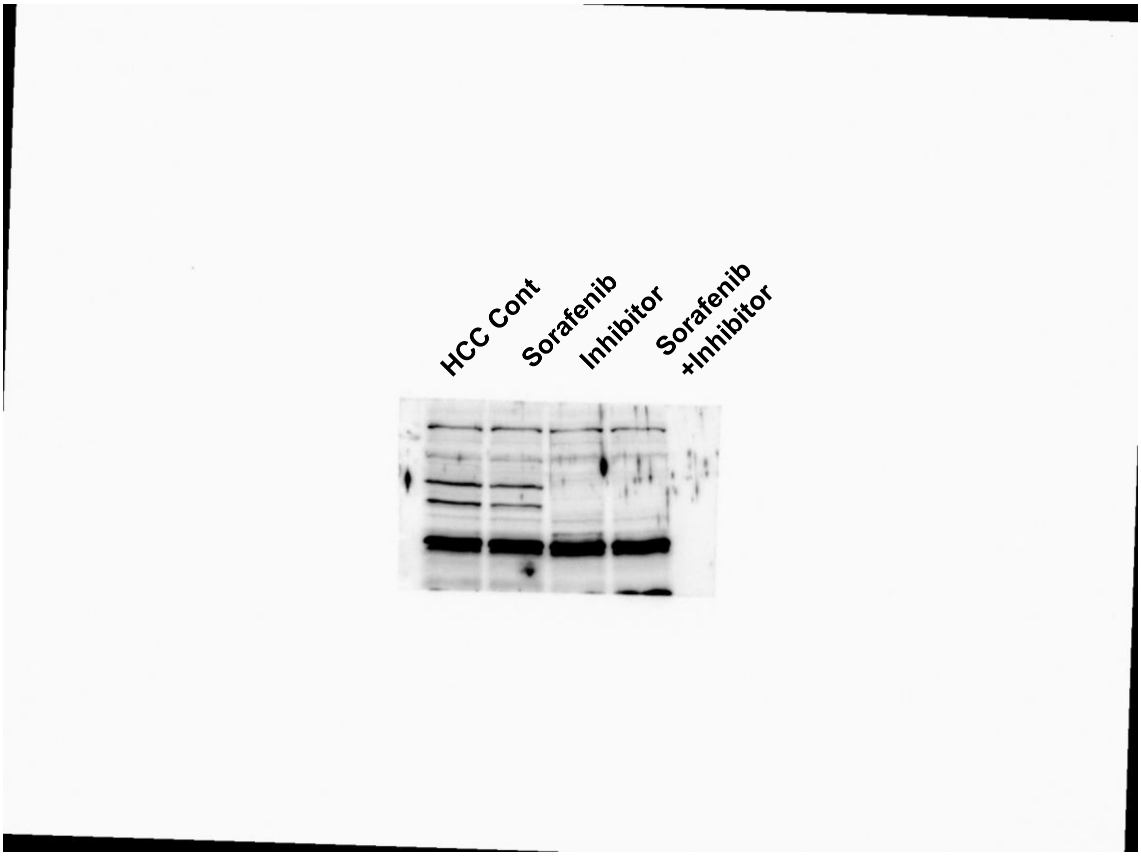

p-AKT

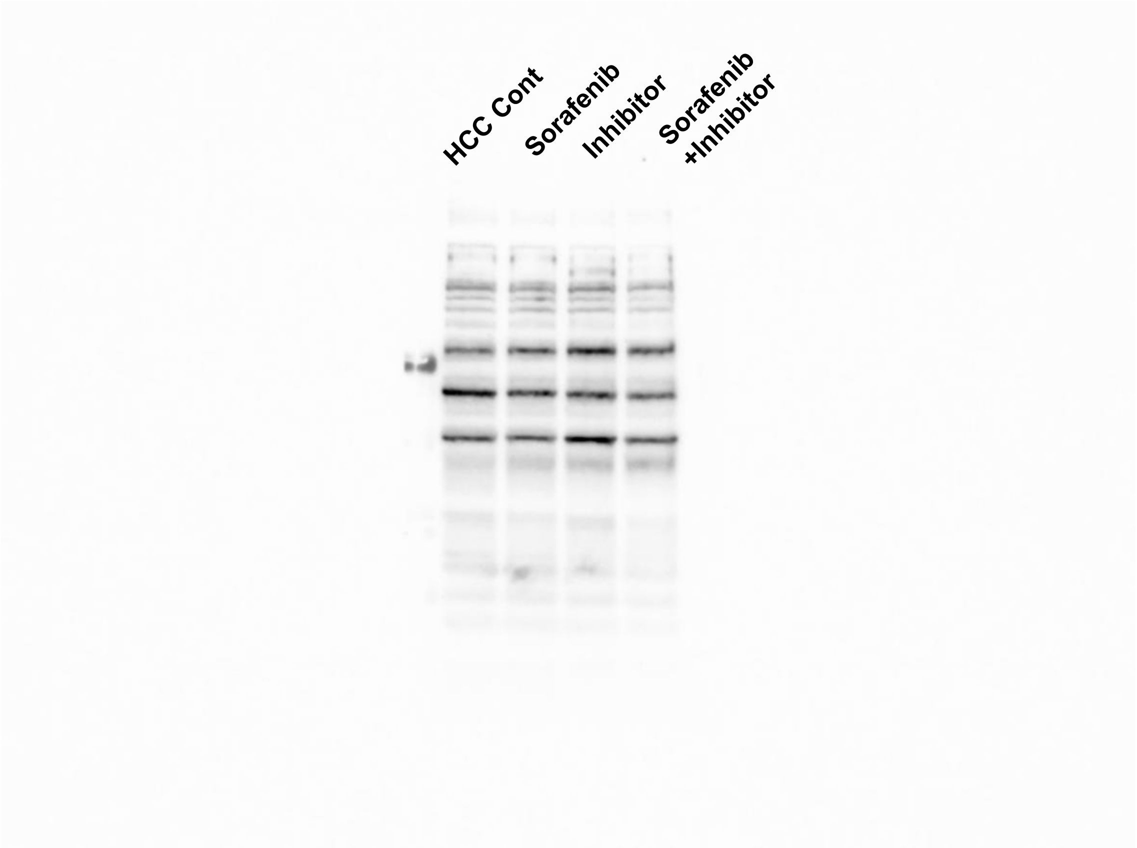

Fig 6

D

NF-κB

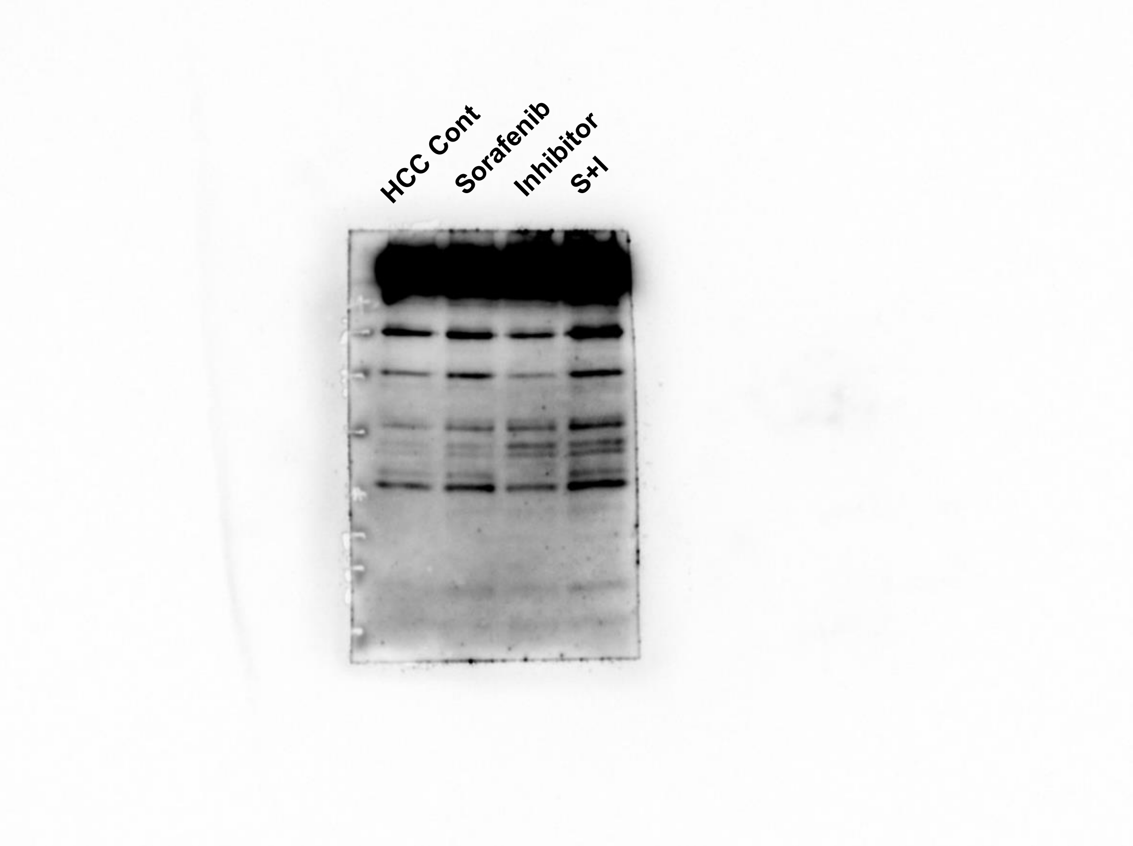

IκB

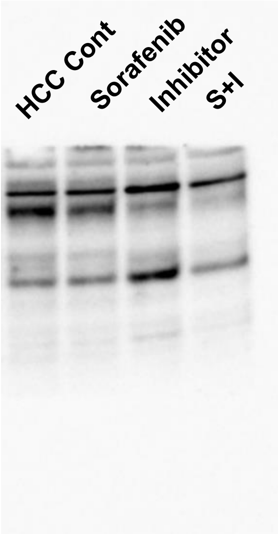

Fig 6

D

Bcl2

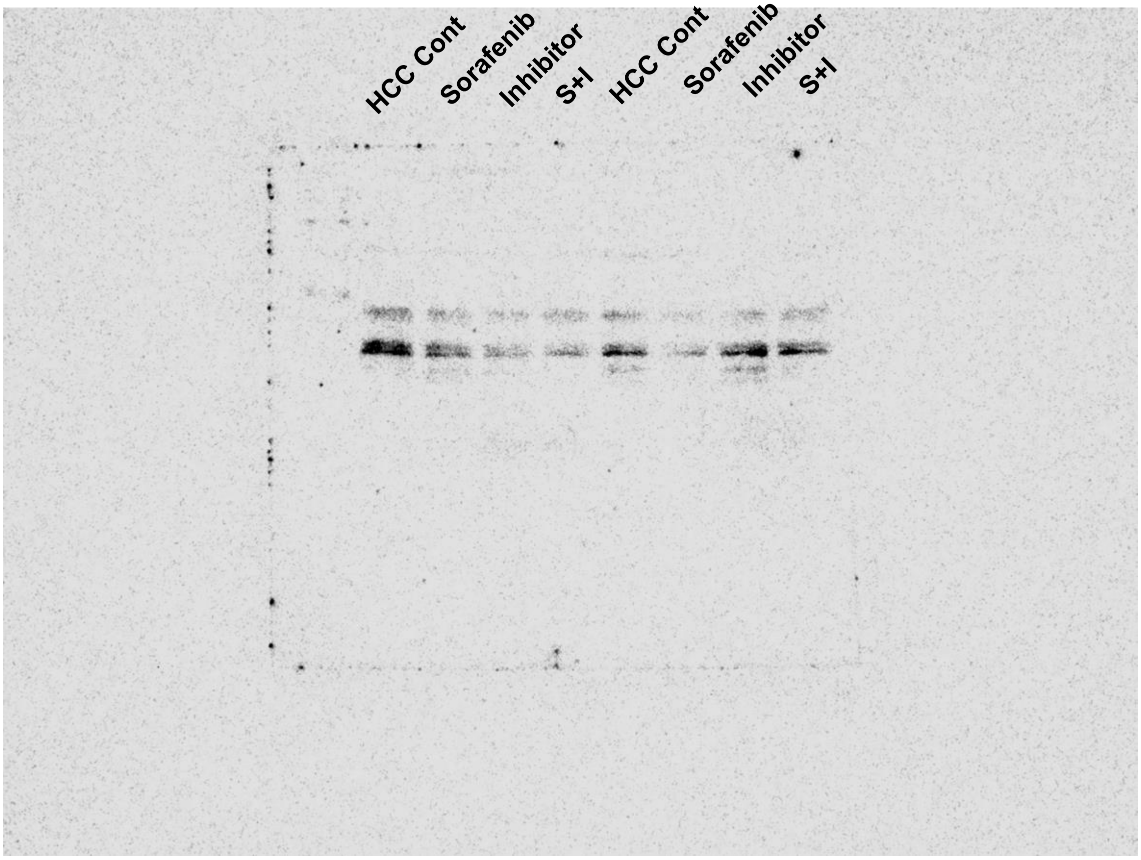

BAX

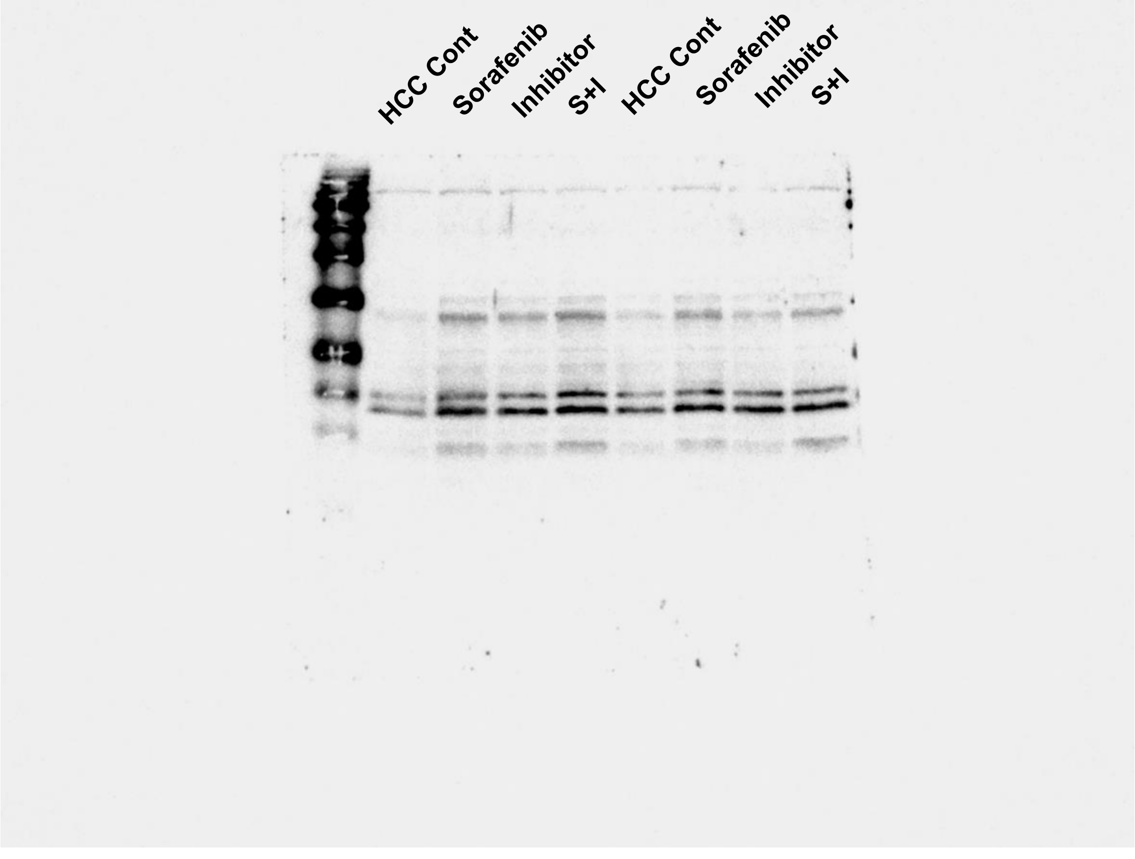

Fig 6

D

β-actin

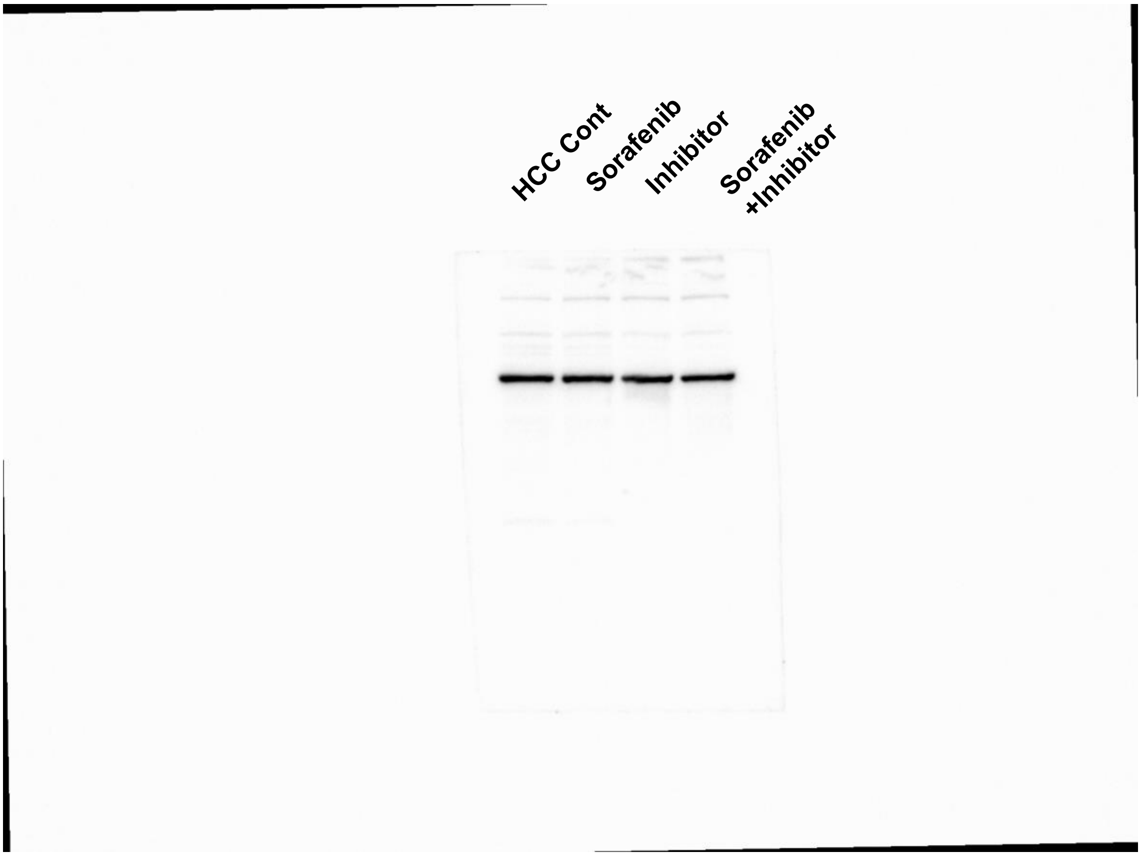

Supplement: Supplementary file 1 [file cancers-18-01038-s001.zip › cancers-4176596-Uncropped Western blot figures.pdf]
